# Health promotion interventions for ethnic minority populations living with multiple long-term conditions (H-PRIME) in OECD countries: a scoping review

**DOI:** 10.1093/heapro/daag147

**Published:** 2026-10-27

**Authors:** Deborah Ikhile, Shabana Cassambai, Shavez Jeffers, Zaynab Randera, Krishna Patel, Brenda Hayanga, Mark P Funnell, Ricky Shah, Riya Patel, Kamlesh Khunti, Angela Harden

**Affiliations:** Diabetes Research Centre, College of Life Sciences, University of Leicester, Leicester General Hospital, Gwendolen Road, Leicester LE5 4PW, United Kingdom; NIHR Applied Research Collaboration East Midlands, Leicester General Hospital, Gwendolen Road, Leicester LE5 4PW, United Kingdom; Diabetes Research Centre, College of Life Sciences, University of Leicester, Leicester General Hospital, Gwendolen Road, Leicester LE5 4PW, United Kingdom; NIHR Applied Research Collaboration East Midlands, Leicester General Hospital, Gwendolen Road, Leicester LE5 4PW, United Kingdom; Centre for Ethnic Health Research, Leicester General Hospital, Gwendolen Road, Leicester LE5 4PW, United Kingdom; Diabetes Research Centre, College of Life Sciences, University of Leicester, Leicester General Hospital, Gwendolen Road, Leicester LE5 4PW, United Kingdom; NIHR Applied Research Collaboration East Midlands, Leicester General Hospital, Gwendolen Road, Leicester LE5 4PW, United Kingdom; Diabetes Research Centre, College of Life Sciences, University of Leicester, Leicester General Hospital, Gwendolen Road, Leicester LE5 4PW, United Kingdom; NIHR Applied Research Collaboration East Midlands, Leicester General Hospital, Gwendolen Road, Leicester LE5 4PW, United Kingdom; Centre for Ethnic Health Research, Leicester General Hospital, Gwendolen Road, Leicester LE5 4PW, United Kingdom; Department of Global Health and Social Medicine, Faculty of Social Science & Public Policy, King’s College London, 40 Aldwych, London WC2B 4BG, United Kingdom; Diabetes Research Centre, College of Life Sciences, University of Leicester, Leicester General Hospital, Gwendolen Road, Leicester LE5 4PW, United Kingdom; NIHR Applied Research Collaboration East Midlands, Leicester General Hospital, Gwendolen Road, Leicester LE5 4PW, United Kingdom; Public Contributor, Leicester, United Kingdom; Diabetes Research Centre, College of Life Sciences, University of Leicester, Leicester General Hospital, Gwendolen Road, Leicester LE5 4PW, United Kingdom; NIHR Applied Research Collaboration East Midlands, Leicester General Hospital, Gwendolen Road, Leicester LE5 4PW, United Kingdom; Centre for Ethnic Health Research, Leicester General Hospital, Gwendolen Road, Leicester LE5 4PW, United Kingdom; Diabetes Research Centre, College of Life Sciences, University of Leicester, Leicester General Hospital, Gwendolen Road, Leicester LE5 4PW, United Kingdom; NIHR Applied Research Collaboration East Midlands, Leicester General Hospital, Gwendolen Road, Leicester LE5 4PW, United Kingdom; Centre for Ethnic Health Research, Leicester General Hospital, Gwendolen Road, Leicester LE5 4PW, United Kingdom; Population Health and Policy, School of Health and Medical Sciences, City St Georges, University of London, Northampton Square, London EC1V 0HB, United Kingdom

**Keywords:** multiple long-term conditions, multimorbidity, health promotion, health inequalities, ethnic minority, socio-ecological model

## Abstract

People living with multiple long-term conditions (MLTC, also called multimorbidity) experience substantial health burden, with those from ethnic minority populations disproportionately affected due to persistent health inequalities. This scoping review identified health promotion interventions designed to address MLTC in ethnic minority populations. Five databases and Google Scholar were searched from inception to June 2025 for studies conducted in the Organization for Economic Co-operation and Development (OECD) countries that included ≥50% ethnic minority participants living with MLTC. Health promotion interventions were defined as programmes, activities, or initiatives delivered in any setting that operate at one or more levels (individual, health system, community, societal, and policy), consistent with the five action areas of the Ottawa Charter for Health promotion. Descriptive and qualitative content analysis was undertaken, with interventions mapped against the Ottawa Charter action areas. Twenty-six studies were included in total, with the majority from the United States of America (USA; *n* = 24, 92%). One study each was conducted in Canada (*n* = 1, 4%) and Australia (*n* = 1, 4%). The interventions aligned with four of the five Ottawa Charter action areas: developing personal skills (26, 100%), creating supportive environments (*n* = 23, 88%), strengthening community action (*n* = 14, 54%), and reorienting health services (*n* = 21, 81%). No intervention directly addressed building healthy public policy. Evidence on health promotion interventions for ethnic minority populations with MLTC is currently underdeveloped, concentrated in the USA, with clear gap in policy action. There is an urgent need for research in OECD countries embedding a multi-level approach to address gaps in policy-actions.

Contribution to Health Promotion

- The burden of multiple long-term conditions (MLTC, also called multimorbidity), is increasing globally.
- People from ethnic minority populations experience higher burden of MLTC because of health inequalities and because some individuals from these populations get certain long-term conditions at an earlier age.
- However, little is known about which health promotion interventions exist for ethnic minority populations living with MLTC.
- This review highlights the need for multi-level approaches (combining individual, interpersonal, organizational, community and policy level) to MLTC care, and greater collaboration with diverse communities in the UK and other OECD countries to design or adapt interventions that are contextually appropriate.

## Introduction

Multiple long-term conditions (MLTC), also referred to as multimorbidity, is defined as the co-existence of two or more long-term conditions in an individual, where no condition is prioritized over others. It has become a global health and care priority in the last decade (The Academy of Medical Sciences, 2018; Skou *et al*. 2022, Khunti *et al*. 2026). It is estimated that more than 14 million people in England are living with MLTC (Valabhji *et al*. 2024), with an increasing MLTC burden observed in low- and middle-income countries (LMICs) (Asogwa *et al*. 2022, Vidyasagaran *et al*. 2024). The global prevalence of MLTC is expected to rise over the coming decades, driven by an increasing ageing population (Chowdhury *et al*. 2023, Khunti *et al*. 2026).

The burden of MLTC is substantial across adult populations across the globe, particularly among older adults, with people living with MLTC reporting poorer quality of life and health outcomes (Hayanga *et al*. 2023, Khunti *et al*. 2023). Evidence from the Organization for Economic Co-operation and Development (OECD) countries shows that socially and economically disadvantaged populations experience a higher burden of MLTC and are more likely to experience adverse health outcomes as a result (Caraballo *et al*. 2022, Valabhji *et al*. 2024, Alvarez-Galvez *et al*. 2026). For example, evidence from both the United Kingdom (UK) and the United States of America (USA) indicates that several ethnic minority populations, including people of Black African, South Asian and Hispanic/Latino descent experience a markedly higher prevalence of MLTC compared with their White counterparts (Caraballo *et al*. 2022, Hayanga *et al*. 2023, Ng Fat *et al*. 2023, Valabhji *et al*. 2024). Notably, MLTC also occurs at younger ages in some ethnic minority populations (Eto *et al*. 2023;Khunti *et al*. 2026). Analysis of the UK Clinical Practice Research Datalink shows that early onset MLTC (before age 40) occurs more frequently among South Asian and Black populations than among White populations (Eto *et al*. 2023). Similar patterns have been observed in the Netherlands, where most ethnic minority populations, including Ghanian, Turkish, and African and South Asian Surinamese experience earlier onset and higher prevalence of MLTC than the Dutch population (Verest *et al*. 2019).

These patterns of earlier onset, coupled with higher prevalence, are mirrored by evidence of poorer outcomes. Compared with White populations, several ethnic minority groups generally experience reduced survival, lower quality of life, and significantly elevated mortality risk associated with MLTC (Stafford *et al*. 2022, NIHR Evidence 2023). Existing evidence further demonstrates that ethnic inequalities in MLTC prevalence and outcomes persist after accounting for differences in age and socioeconomic disadvantage, indicating that factors beyond demographic and socioeconomic characteristics contribute to these inequalities (Verest *et al*. 2019, Ng Fat *et al*. 2023). Together, this growing body of evidence highlights the profound and persistent inequalities experienced by ethnic minority populations.

Despite increasing recognition of the scale and complexity of the challenges of MLTC, contemporary health systems remain inadequately equipped to respond effectively to MLTC, largely due to their continued orientation towards single disease models of care, compounded by workforce shortages, fragmented service delivery, and rising demand (Skou *et al*. 2022, Khunti *et al*. 2025). Addressing MLTC therefore requires a holistic, system-wide approach that situates individuals within the broader dynamic and interdependent social, economic, and environmental contexts that shape their health trajectories.

Within this context, a health promotion perspective is pivotal, not only for preventing the progression of MLTC across the life course, but also for tackling the structural inequalities that drive their unequal prevalence and outcomes. The World Health Organization (WHO) defines health promotion as enabling people to gain greater control over their health through improved health literacy and coordinated efforts across sectors (World Health Organization 2015). The Ottawa Charter for Health Promotion (1986) further positions it across five action areas: building healthy public policy (policy level), creating supportive environments (societal level), reorientating healthcare services (health system level) community action (community level), developing personal skills (individual level); highlighting its relevance to complex conditions such as MLTC.

Despite its relevance, there is currently no review that synthesizes research on MLTC interventions from a health promotion perspective with a specific focus on ethnic minority populations. A global umbrella review published in 2023 identified 30 eligible reviews comprising 464 unique studies on MLTC interventions (Zhou *et al*. 2023), yet none of the included studies examined outcomes for ethnic minority groups. Similarly, a systematic review published in 2024, focused on North America and Europe highlighted the under-representation of ethnic minority populations in MLTC research (Kayani *et al*. 2024). This scoping review therefore aimed to identify and map existing research on MLTC-related health promotion interventions implemented at individual, health system, community, societal and policy levels, with particular attention to ethnic minority populations. To achieve this aim, the review was guided by the following questions:

1. What types of health promotion interventions have been described in the literature to address multiple long-term conditions (MLTC) in ethnic minority populations?
2. Which Ottawa Charter action areas are reflected in these interventions, and in what settings are they delivered?
3. What individual, health system, community, societal, and policy-level outcomes have been reported?
4. Which theories and frameworks underpin the health promotion interventions?
5. What evidence gaps exist in the current MLTC and ethnic inequalities literature?

## Methods

This scoping review followed the Johanna Briggs Institute (JBI)’s guidance for scoping reviews (Peters *et al*. 2015) and the Preferred Reporting Items for Systematic Reviews and Meta-Analysis extension for Scoping Reviews (PRISMA-ScR) (Tricco *et al*. 2018) (Supplementary File S1). This review was originally planned as a systematic review to assess the effectiveness of health promotion interventions for MLTC among ethnic minority populations, with a protocol prospectively registered on PROSPERO (CRD42024528790). However, following advice from a scientific committee, the protocol was revised to conduct a scoping review in the first instance to map the existing evidence. The revised protocol for the scoping review was subsequently registered on Figshare repository (Ikhile *et al*. 2025). The review was conducted according to the published scoping review protocol. One deviation from the protocol was the revision of review questions to better align with the aim.

### Eligibility criteria

Eligibility criteria were defined using the JBI’s population, concept, and context framework (Peters *et al*. 2015). Broadly, in relation to population, eligible studies included individuals from ethnic minority groups, as defined within the primary studies, living with MLTC (i.e. two or more long-term conditions). A global Delphi consensus study conducted in 2022 provides a list of 24 core or 35 optional conditions to include in MLTC research, which was used to determine eligible conditions (Ho *et al*. 2022). Given the heterogeneity in MLTC definitions and conflation with other terms such as comorbidity (Khunti *et al*. 2023, Funnell *et al*. 2026), studies focusing on comorbidity were also included where an index condition was specified and at least 70% of participants had additional conditions, a threshold used in previous MLTC reviews (Ikhile *et al*. 2026).

In terms of concept, health promotion interventions were defined as programmes, activities, or initiatives delivered in any setting, and operating at an individual, health system, community, societal or policy level to enable and promote healthy behaviours, consistent with the WHO definition and Ottawa Charter action areas (Ottawa Charter for Health Promotion 1986, World Health Organization 2015). The context was restricted to the OECD countries to reduce contextual heterogeneity and enhance interpretability by focusing on settings with relatively developed healthcare systems, established health data infrastructures, and similar epidemiological challenges related to MLTC. While variation exists between OECD member states, this approach enabled a focused synthesis of evidence on ethnic inequalities in MLTC and improved the relevance of findings to the UK context.

Eligible studies were primary studies of any design (quantitative, qualitative, and mixed methods) evaluating health promotion interventions for MLTC in ethnic minority populations. Studies were eligible when ≥50% of the study population comprised ethnic minority participants, as reported in the primary article.

Grey literature, case reports, theses, reviews, book chapters, conference proceedings, and posters were excluded, as were studies from which relevant participant or intervention characteristics (including number and type of conditions) could not be extracted. Non-English-language publications were also excluded. The full eligibility criteria are detailed in Supplementary File S2.

### Study identification

Five relevant electronic databases, MEDLINE, the Cochrane Library, CINAHL, Scopus, PsycINFO, and Google Scholar were searched from their respective inception date to 27 January 2025. All records identified through Google Scholar were retrieved, and the platform was additionally used to conduct manual searches. Search terms were guided by the PPC framework and used free-text terms and database-specific subject headings, with strategies tailored to each database using Boolean operators. The initial search was conducted by DI and was supplemented by manual screening of reference lists from included studies and relevant systematic reviews. This was further complemented by hand-searched materials from another MLTC review conducted by one member of the review team (B.H.) in May 2025 (Hayanga *et al*. 2026). An updated search for all five databases was conducted on 30 June 2025, with ongoing manual search on Google Scholar until 30 October 2025. The search strategy was informed by prior patient and public involvement and engagement (PPIE) conducted in 2024 with a community group comprising Black African and Black Caribbean participants (Ikhile 2025). It was refined with support from an Information Specialist to ensure alignment with database-specific requirements. All search strategies are provided in Supplementary File S2.

### Study selection

Following the search, all identified citations were downloaded into EndNote 20 and then uploaded on COVIDENCE software™ for de-duplication and screening. One reviewer (D.I.) independently screened all records during both the title and abstract and full text screening stages. To ensure that all records were double-screened, each article was also independently reviewed by a second member of the team (S.C., M.P.F., S.J., and Z.R.), with each reviewer screening ∼25% of the records. Conflicts arising during the screening phase were resolved through discussion and consensus.

### Data charting

A data charting template was developed and piloted in Microsoft Excel. Five reviewers were involved in the data extraction (DI, SC, ZZ, SJ and MF), with all data extracted independently by two reviewers to ensure consistency and rigour. Any conflicts arising at this stage were resolved by discussion and consensus. Extracted data included: author details, publication year, country, study aim, study type, participants characteristics, MLTC definition and conditions included, intervention description, outcomes and key findings. Intervention description was guided by the template for intervention description and replication checklist, where applicable, while participant characteristics were determined according to protected characteristics and guided by an equality impact assessment framework (NIHR Applied Research Collaboration Eastmidlands 2025).

### Patient and public involvement and engagement

Four PPIE sessions (two in-person, two virtual PPIE) were held with members of the public from Black African, Caribbean, and South Asian backgrounds who were either living with MLTC or caring for someone with MLTC, between May and November 2025. PPIE meetings occurred after data extraction and aimed to ensure the relevance, and cultural and contextual appropriateness of the review findings by incorporating lived experiences into the interpretation of findings. In addition, a co-production workshop was held in November 2025 after data analysis as part of a community-based dissemination event and aimed to co-produce recommendations for future action with members of the public, community leaders, and MLTC researchers.

### Ethical consideration and equality impact assessment

Ethical approval was not required for this study as it was a scoping review of published literature and did not involve the collection of primary research data. The PPIE activities similarly did not require ethical approval, as they were conducted to inform and enhance the review process rather than generate research data. Nevertheless, informed consent was obtained from all PPIE participants, and no identifiable personal information was collected or recorded. The review was conducted in accordance with established methodological guidance for scoping reviews. Also, to ensure representativeness and meaningful engagement, an equality impact assessment (NIHR Applied Research Collaboration Eastmidlands 2025) was conducted prior to the start of the scoping review. The equality impact assessment ensured diverse individuals were involved and was used to ensure key sociodemographic variables in line with the protected characteristics of the UK Equality Act 2010 were extracted.

### Data analysis and presentation

Descriptive and qualitative content analysis approaches were employed to summarize and interpret the extracted data. Study and participant characteristics were analysed descriptively using frequency counts and presented in tables and diagrams, where appropriate.

Qualitative content analysis was applied to examine intervention components and key findings from the included studies. Interventions were inductively grouped into overarching categories and outcomes were classified into relevant domains. A subsequent deductive phase of analysis mapped the identified interventions to the five Ottawa Charter action areas: (i) building healthy public policy; (ii) creating supportive environments; (iii) strengthening community action; (iv) developing personal skills; and (v) reorienting health services. The mapping criteria are provided in Supplementary File S3. D.I. led the analysis, which was subsequently reviewed by M.P.F. and R.P., and discussed with them to enhance rigour and minimize potential bias in interpretation. Preliminary findings were also presented to the PPIE group, and members enriched the analysis by contributing their lived experiences and contextual perspectives. The PPIE group further provided recommendations on the presentation of findings and on priorities for future research. Final findings were shared through a community dissemination and co-production workshop. Insights generated during the workshop, together with contributions from separate PPIE meetings, were synthesized narratively and creatively represented through a visual illustration.

## Results

A total of 11 677 records were identified from the database, manual and complementary searches. After removing 2861 duplicates, 8816 records remained for title and abstract screening. Of these, 8456 were excluded, leaving 360 for full-text screening. Following full-text screening, 26 studies met the inclusion criteria and were included in this review. The study selection process is outlined in the PRISMA flowchart (Fig. 1).

**Figure 1:**
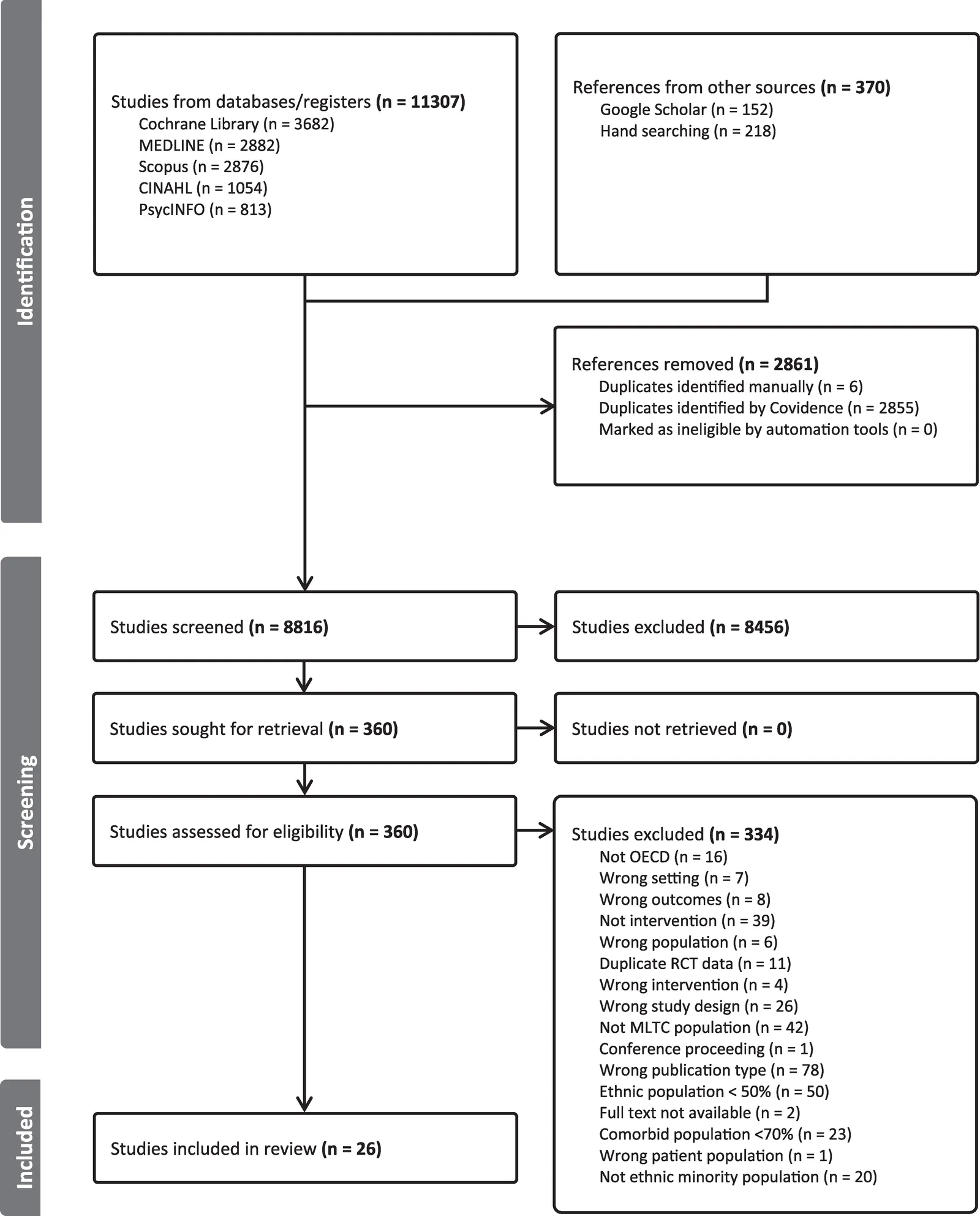
PRISMA flowchart.

### Study characteristics

Twenty-four studies (92%) were conducted in USA, with one study in Australia (4%) and Canada (4%). The most common study design was randomized controlled trial (*n* = 18, 69%), followed by feasibility or pilot studies (*n* = 8, 31%). Sample size varied considerably, ranging from 8 in a community-based pilot study (Breslow *et al*. 2024) to 801 in a hospital-based randomized controlled trial (Metsch *et al.* 2016), with a median sample size of 147 participants. Studies were published between 2002 and 2024, with the highest number published in 2024 (*n* = 5, 19%).

Of the 26 included studies, 14 recruited participants from a single ethnic minority group. Hispanic/Latino populations were the most frequently represented (*n* = 7, 27%), followed by African American/Blacks (*n* = 5, 19%), South Asian (*n* = 1, 4%) and First Nations (*n* = 1, 4%). The remaining 12 (46%) studies included participants from a range of ethnic groups (Table 1). There was notable variation in the reporting of participants characteristics. For example, several studies conducted in the USA reported both race and ethnicity. Where available, ethnicity data was prioritized and extracted; otherwise, race was used as a proxy for inclusion. In the study conducted in Australia, ethnicity was determined by the primary language spoken by participants (Williams *et al*. 2012). Overall, studies generally had a larger proportion of women (median = 64%, range = 23%–100%), and where reported, mean age ranged from 44.6 to 74.3 years. Other sociodemographic data (marital status, religious belief, and socioeconomic status) were inconsistently reported across studies. Full study characteristics are provided in Table 1.

**Table 1:** Study characteristics.

First author, year	Aim	Country	Study design	Participants	Ethnicity	Sex	Mean age
**Aharonovich *et al*. 2017**	To investigate the feasibility and efficacy of reducing non-injection drug and alcohol use with Motivational interviewing + HealthCall as adapted for smartphone technology.	USA	Randomized pilot trial	Urban minority adults living with HIV with co-occurring substance/drug use (*N* = 47)	African American/Black: 78.7% Hispanic: 17.0% Other: 4.3%	F: 23% M: 77%	50.9
**Alexopoulos *et al*. 2024**	To evaluate the feasibility and acceptability of a telehealth intervention that delivers all components of Evidence-based management of MASLD in Latino/a and Black patients with type 2 diabetes.	USA	Feasibility pilot study	Latino/a and Black patients with type 2 diabetes and metabolic dysfunction-associated steatotic liver disease (MASLD) (*N* = 10)	African American/Black: 60.0% Latino/a: 40.0%	F: 50% M: 50%	51.1
**Bogner and de Vries 2008**	To examine whether integrating depression treatment into care for hypertension improved adherence to antidepressant and antihypertensive medications, depression outcomes, and BP control among older primary care patients.	USA	Pilot randomized controlled trial	Older adults (50–80 years) prescribed pharmacotherapy for depression and hypertension (*N* = 64)	African American/Black: 81.2% White: 17.2%	F: 76.6% M: 23.4%	58.6
**Breslow *et al*. 2024**	To address depression using a therapeutic approach targeting gender-specific processes and internalized stigma among a small pilot group of older women living with HIV.	USA	Community-led pilot study (mixed methods)	Women, 55 years and older living with HIV and experiencing past-year depressive symptoms (*N* = 8)	African American/Black: 62.5% Hispanic/Latino/a: 37.5%	F: 100%	59.5
**Diamantidis *et al*. 2018**	To evaluate the effectiveness of a multi-factorial behavioural and medication management intervention to mitigate kidney function decline at 3 years compared with usual care.	USA	Randomized controlled trial	Adults with diabetes and uncontrolled hypertension and evidence of kidney dysfunction (*N* = 281)	Non-Hispanic white: 41.28% Non-Hispanic Black: 55.52% Asian: 0.71% American Indian/Alaska Native: 0.36% Other/Hispanic: 2.14%	F: 48.4% M: 51.6%	61.9
**Dunbar *et al*. 2015**	To test an integrated self-care intervention on outcomes of heart failure and diabetes quality of life.	USA	Randomized controlled trial	Adults with heart failure and diabetes mellitus (*N* = 134)	African American: 69.4%	F: 34.3% M: 65.7%	57.4
**Eakin *et al*. 2007**	To evaluate a physical activity and diet intervention targeting low-income, largely Spanish-speaking patients with multiple chronic conditions.	USA	Randomized controlled trial	Low-income, predominantly Spanish-speaking urban Latino adults with multiple chronic conditions for which a lifestyle intervention focused on physical activity and diet would be appropriate (*N* = 200)	Hispanic/Latino: 75% Anglo: 15% Other: 9%	F: 78.5% M: 21.5%	50
**Ell *et al*. 2010**	To determine whether evidence-based socio-culturally adapted collaborative depression care improves receipt of depression care and depression and diabetes outcomes in low-income Hispanic subjects.	USA	Randomized controlled trial	Low-income Hispanic patients with major depression and diabetes (*N* = 387)	Hispanic: 96% non-Hispanic: 4%	F: 82.0% M: 18.0%	NR
**Ell *et al*. 2017**	To evaluate depression and self-care management among patients with diabetes and/or heart disease in a 12-month randomized trial.	USA	Randomized controlled trial	Adult patients with depression and concurrent chronic condition (diabetes = 96%, and/or heart disease = 4%) (*N* = 348)	Hispanic/Latino: 99%	F: 85.0% M: 15.0%	56.54
**Feldman *et al*. 2016**	To compare cognitive behaviour psychophysiological therapy to music and relaxation therapy on panic disorder severity, asthma control, and several other anxiety and asthma outcome measures.	USA	Randomized controlled trial	Latino adults with asthma and panic disorder (*N* = 53)	Puerto Rican: 81% Other Latino: 19%	F: 94.0% M: 0.6%	NR
**Gallo *et al*. 2023**	To test the effectiveness of a culturally appropriate care transitions programme for Latino adults with multiple cardiometabolic conditions and behavioural health concerns in reducing hospital utilization and improving patient-reported outcomes.	USA	Randomized controlled trial	Latino adults with multiple cardiometabolic conditions and behavioural health concern (e.g. depressive symptoms, alcohol misuse) (*N* = 536)	Hispanic/Latino: 100%	F: 48.0% M: 52.0%	62.3
**Gardiner *et al*. 2019**	To determine the effectiveness of Integrative Medical Group Visits (IMGV) compared with a Primary Care Provider (PCP) visit in patients with chronic pain and depression.	USA	Randomized controlled trial	Predominantly low-income racially diverse adults with non-specific chronic pain and depressive symptoms (*N* = 159)	Ethnicity Hispanic:14% Non-Hispanic: 86%* Race White: 19% Black: 56% Multiple race: 6% Unknown/not reported: 30%	F: 86.0% M: 14.0%	50.5
**Jean-Louis *et al*. 2017**	To assess effectiveness of a culturally and linguistically tailored telephone-delivered intervention to increase adherence to physician-recommended evaluation and treatment of obstructive sleep apnoea (OSA) among Blacks.	USA	Randomized controlled trial	Blacks with metabolic syndrome and sleep apnoea (*N* = 303)	Black: 100%	F: 71.3% M: 28.7%	NR
**Jones *et al*. 2024**	To evaluate the efficacy of a behavioural pain self-management intervention called STOMP compared with enhanced usual care (EUC).	USA	Randomized controlled trial	Adults with HIV and chronic pain (*N* = 278)	Ethnicity Hispanic:1% Non-Hispanic: 99%* Race White: 17% Black/African American: 81% Other or American Indian/Alaska native: 3%	F: 44.0%, Transgender F: 2.0% M: 53.0%	53.5
**Kangovi *et al*. 2017**	To determine whether a CHW intervention improved outcomes in a low-income population with multiple chronic conditions.	USA	Randomized controlled trial	High-poverty neighbourhood residents, uninsured or publicly insured, and diagnosed with ≥2 chronic conditions (diabetes, obesity, hypertension, tobacco dependence) (*N* = 302)	African American: 94.7% Hispanic: 2.7%	F: 75.5% M: 24.5%	56.1
**Lynch *et al*. 2014**	To determine effectiveness of an intensive community based, group intervention focused on diet, PA, peer support for reducing weight in urban dwelling African Americans with type 2 diabetes and hypertension.	USA	Pilot randomized controlled trial	Low-income African American adults with type 2 diabetes and hypertension (*N* = 61)	African American: 100%	F: 67.2% M: 32.8%	54.1
**Metsch *et al*. 2016**	To assess the effect of a structured patient navigation intervention with or without financial incentives to improve HIV-1 viral suppression rates among patients with elevated HIV-1 viral loads and substance use recruited as hospital inpatients.	USA	Randomized controlled trial	Hospitalized adults with HIV infection and substance use (*N* = 801)	Race/Ethnicity* Hispanic: 11.1% Black: 77.5% White: 19.1% Other: 4.8%	F: 32.6% M: 67.4%	44.6
**Metsch *et al*. 2021**	To test the efficacy of a 6-month care facilitation intervention.	USA	Randomized controlled trial	Adults with HIV and hepatitis C virus (HCV) coinfection and active substance use (*N* = 113)	Hispanic/Latino: 14.1% Black or African American: 72.6% White: 12.4%	F: 41.6%k M: 58.4%	51
**Nuño *et al*. 2023**	To assess the feasibility and outcomes of a behavioural intervention to decrease CVD and complications in a Hispanic diabetic population.	USA	Pilot feasibility study	Hispanic adults with type-2 diabetes, at high risk for cardiovascular disease (*N* = 30)	Hispanic: 100%	F: 70.0% M: 30.0%	61
**Ogedegbe *et al*. 2024**	To determine whether nurse case management plus Home BP Telemonitoring (HBPTM) results in greater systolic BP (SBP) reduction at 12 months and lower rate of stroke recurrence at 24 months than HBPTM alone among Black and Hispanic stroke survivors with uncontrolled hypertension.	USA	Randomized controlled trial	Black and Hispanic adults with prior stroke and uncontrolled hypertension (*N* = 450)	Race/Ethnicity* Black, non-Hispanic: 51.0% Hispanic: 49.0%	F: 44.4% M: 55.6%	61.7
**Rimmer *et al*. 2002**	To determine if a group of predominantly low-income, low-education, African American women with type 2 diabetes could achieve good compliance and improved health outcomes with a carefully structured health promotion intervention.	USA	Feasibility study	African American, Asian American, Hispanic, White with type 2 diabetes (*N* = 30) and high BP (75.9%)	African American: 80.0% Asian American: 3.3% Hispanic: 6.6% White: 10.0%	F = 100.0%	54.9
**Shah *et al*. 2024**	To test the effectiveness of an evidence-based CHW-led and culturally tailored HTN and DMII management programme for South Asian adults with diabetes and comorbid uncontrolled hypertension.	USA	Randomized controlled trial	South Asian immigrants with type 2 diabetes and comorbid hypertension (*N* = 190)	South Asians: 100.0%	F = 56.3% M = 43.7%	56
**Tobe *et al*. 2006**	To assess whether a community-based treatment strategy implemented by home care nurses would be effective in controlling hypertension in First Nations people with existing hypertension and type 2 diabetes.	Canada	Randomized controlled trial	First Nations adults with existing hypertension and type 2 diabetes (*N* = 100)	First Nations = 100.0%	F: 61.5% M: 38.5%	NR
**Towfighi *et al*. 2021**	To determine if a chronic care model–based, CHW, advanced practice clinician (APC; including nurse practitioners or physician assistants), and physician team intervention improves risk factor control after stroke in a safety-net setting (i.e. health care setting where all individuals receive care, regardless of health insurance status or ability to pay).	USA	Randomized controlled trial	Adults ≥40 years with recent ischemic or haemorrhagic stroke or transient ischemic attack (≤90 days) and uncontrolled hypertension (*N* = 487)	Hispanic: 71.3%	F = 34.9% M = 65.1%	57
**Williams *et al*. 2012**	To test the feasibility and impact of a multi-factorial Self-efficacy medication (SEM) intervention to improve medication self-efficacy and adherence for Culturally and linguistically diverse (CALD) consumers with chronic kidney disease, diabetes and cardiovascular disease.	Australia	Randomized controlled trial	Adults (≥18 years) with chronic kidney disease, diabetes, and cardiovascular disease from culturally and linguistically diverse (CALD) groups (Greek, Italian, Vietnamese speakers) (*N* = 78)	Country of birth^b^ Greece: 33.3% Italy: 48.0% Vietnam: 16.7% Other (Egypt but Greek upbringing): 2.1%	F: 70.0% M: 30.0%	74.3
**Zuñiga *et al*. 2019**	To adapt a community-based diabetes self-management intervention for people living with HIV and test the feasibility of administering the intervention with persons living with HIV + type 2 diabetes who are low-income, predominantly minority, vulnerable population.	USA	Feasibility trial	Adults (≥18 years) living with HIV and type 2 diabetes (*N* = 25)	Race/Ethnicity^a^ White: 30.8% African American: 58.7% Latino: 19.2% Other: 7.7%	F: 24.0% M: 68.0%	56.6

USA, United States of America.

^a^Denotes studies that did not report a breakdown of the non-Hispanic population, or that did not distinguish between ethnicity and race.

^b^Denotes participant categorization based on Country of birth and language spoken (Greek, Vietnamese, or Italian).

Regarding condition combinations, studies used a range of terminologies to define MLTC, including multiple chronic conditions, concomitant, co-occurring, comorbidity, multimorbidity, and concurrent. Diabetes, hypertension and HIV were the most included conditions (Supplementary File S4), often in combination with depression or cardiovascular disease. One exception was a study focused explicitly on MLTC prevention, which included participants with type 2 diabetes at high risk of cardiovascular disease (Nuño *et al*. 2023).

### Intervention characteristics

Intervention characteristics are detailed in Supplementary File S5. Across the 26 included studies, each study evaluated one intervention. Interventions clustered into four broad categories: (i) collaborative and integrated care models, (ii) task-shifting interventions, (iii) self-management education and skills-building interventions, and (iv) technology-enabled monitoring and telehealth interventions. Some interventions were adaptations of existing evidence-based approaches, often culturally tailored to the target population, rather than entirely novel interventions.

Most of the 26 interventions did not report any underpinning theory or framework (*n* = 16, 62%). Seven studies (27%) reported using cognitive-behavioural therapy (CBT) (Lynch *et al*. 2014, Feldman *et al*. 2016), transtheoretical model of change (Jean-Louis *et al*. 2017), social cognitive theory (Jones *et al*. 2024), health belief model (Williams *et al*. 2012, Towfighi *et al*. 2021, Shah *et al*. 2024), and social support theory (Shah *et al*. 2024). Three frameworks were reported across 4 studies (15%): the ADAPT-ITT framework used in the electronic stress management and relaxation training/expressive supportive therapy (e-SMART/EST) for women living with HIV and depression (Breslow *et al*. 2024), PRECEDE model used in the Metso trial focusing on Black adults with Metabolic Syndrome and Sleep Apnoea (Jean-Louis *et al*. 2017), and socio-ecological model used in the Resources for Health intervention for Latino adults living with multiple chronic conditions (Eakin *et al*. 2007) and *Mi Puente* intervention for Latino adults living with multiple cardiometabolic conditions (Gallo *et al*. 2023).

Intervention duration varied substantially, ranging from 4 weeks to 3 years, while follow-up periods ranged from 6 weeks to 3 years. Primary care/community (*n* = 7, 27%) and hospitals (*n* = 12, 46%) were the dominant delivery settings, reflecting their central role in the management of MLTC. Six interventions (23%) were delivered virtually and one (4%) was delivered in a university setting. Most interventions used hybrid delivery models, combining in-person and remote components, with increasing use of digital health strategies, particularly in studies published after the COVID-19 pandemic.

Across the 26 included studies, primary and secondary outcomes spanned multiple domains, clinical, behavioural and psychosocial, health-related quality of life, implementation, social determinants, health services and economic domains. Clinical and biomedical outcomes were assessed in the majority of studies (*n* = 20, 77%), for example, by measurement of blood pressure (BP), glycaemic control, HIV viral suppression, pain severity, and asthma control. Mortality outcomes were only assessed in two studies (8%). Similarly, 20 studies (77%) evaluated behavioural, and psychosocial outcomes, including medication adherence, patient activation, self-efficacy, lifestyle behaviours, disease knowledge, and social support. Health-related quality of life was also commonly assessed, with 12 studies (46%) using generic or condition-specific measures, such as the 12-Item Short Form Health Survey (SF-12), the EuroQol 5-Dimension Questionnaire, the Medical Outcomes Study HIV Health Survey (MOS-HIV SF-12), the Minnesota Living with Heart Failure Questionnaire, Mini Asthma Quality of Life Questionnaire, and the Audit of Diabetes-Dependent Quality of Life, alongside physical functioning or activity assessments. Implementation outcomes were explicitly reported in six studies (23%), encompassing feasibility, acceptability, appropriateness, fidelity, and recruitment and retention metrics, often using standardized measures or Likert scales. Only one RCT used the RE-AIM framework. Six studies (23%) reported social determinants of health outcomes as secondary measures, specifically socioeconomic stress, social and instrumental support, structural barriers to care, or access-related factors. Only one (4%) of the 26 included studies incorporated explicit economic outcomes, conducting cost-effectiveness analyses at 12 and 24 months (Ogedegbe *et al*. 2024). Although some studies reported healthcare utilization outcomes, these were not accompanied by cost analyses and were therefore classified as health services outcomes (*n* = 6, 23%) rather than economic endpoints.

#### Collaborative and integrated care models

Ten studies (38%) employed collaborative and integrated care models as the approach for managing MLTC. These interventions explicitly aimed to coordinate care across conditions, providers, or sectors, often combining medical management with behavioural health, care coordination, or navigation support. Another core component of these interventions was the use of multidisciplinary approaches and shared responsibility across multidisciplinary teams including primary care clinicians, care managers, mental health specialists, advanced practice clinicians, and, in some cases, community health workers (CHWs).

Collaborative and integrated care interventions integrating mental and physical health management demonstrated improvements in both biomedical and psychosocial outcomes. For example, Bogner and de Vries (2008) pilot RCT on integrated pharmacologic management for older adults (50–80 years) with depression and hypertension reported improved medication adherence, BP control, and depressive symptoms. The multifaceted diabetes and depression programme among low-income Hispanic patients with major depression and diabetes also showed sustained depression improvement and better functioning in a RCT among low-income Hispanic adults with diabetes and depression, using a culturally adapted collaborative care model (Ell *et al*. 2010).

Integrated care models addressing two physical conditions showed more mixed effects. The integrated heart failure and diabetes self-care intervention improved heart failure related quality of life but not diabetes specific outcomes in a RCT (Dunbar *et al*. 2015). More complex care transition and navigation-focused interventions also featured in this category. For example, Gardiner *et al*. (2019) RCT evaluated integrative medical group visits for chronic pain and depression in low-income racially diverse adults, but found no significant improvements in primary outcomes, although reductions in emergency department use and pain medication were observed. Gallo *et al*. (2023) RCT also evaluated *Mi Puente*, a culturally tailored inpatient-to-outpatient integrated care transitions programme for Latino adults with multiple cardiometabolic conditions and behavioural health concerns found no reductions in readmissions compared with usual care. Similarly, patient navigation based integrated interventions among people with HIV and substance use improved engagement along care continua but did not translate into viral suppression or clinical cure (Metsch *et al*. 2016, 2021).

#### Task-shifting models

Five studies (19%) evaluated task-shifting interventions in which defined elements of chronic disease management, behavioural support, or care coordination were led by non-physician providers, including nurses, pharmacists, CHWs and patient navigators. Although elements of task shifting are embedded in collaborative care models, interventions presented here are those primarily designed to be led by non-physicians.

Nurse-led and pharmacist-led interventions were generally feasible but produced mixed clinical effects. For example, Ogedegbe *et al*. (2024) RCT found that nurse case management combined with home BP telemonitoring led to significantly greater systolic BP reductions than telemonitoring alone in Black and Hispanic adults with prior stroke and uncontrolled hypertension. In contrast, nurse-directed hypertension management among First Nations adults with hypertension and type 2 diabetes did not outperform usual physician-directed care on systolic BP outcomes in a RCT (Tobe *et al*. 2006).

In contrast, CHW models focused more on culturally appropriate engagement, navigation, behavioural support, and addressing social barriers, and demonstrated high engagement and positive primary outcomes. For example, Kangovi *et al*. (2017) RCT demonstrated that individualized CHW support in the IMPaCT intervention significantly improved control of multiple chronic conditions in high-poverty neighbourhood residents. Similarly, among South Asian immigrants with diabetes and hypertension, a CHW-led telehealth RCT achieved significantly greater improvements in BP control and modest weight loss, alongside high completion and retention rates (Shah *et al*. 2024). However, A Helping Hand *promotoras* intervention did not yield significant outcomes in comparison to usual care in a RCT with adults living with depression and a physical chronic condition (Ell *et al*. 2017). It was noted that this was likely due to enhancement of the usual care following the Affordable Care Act enactment and expansion of Medicaid during the study period (Ell *et al*. 2017).

#### Self-management education and skills-building interventions

Eleven studies (42%) focused primarily on self-management interventions enhancing patients’ skills, confidence, and behaviours related to chronic disease management, often through education, behavioural training, and peer or provider support. Core components typically combined education with behavioural skill development, including goal setting, problem solving, motivational interviewing, mindfulness, or cognitive-behavioural techniques. Delivery formats included group-based sessions, individual counselling, telephone delivery, and written or digital materials.

Across studies, self-management interventions showed improvement in behavioural and psychosocial outcomes, including self-efficacy, coping skills, medication adherence, and health behaviours. For example, the adapted diabetes self-management feasibility trial for people living with HIV demonstrated significant improvements in diabetes self-management skills, although changes in biomedical markers were limited (Zuñiga *et al*. 2019). Lifestyle interventions, such as Lifestyle Improvement through Food and Exercise in low-income African American adults with type 2 diabetes and hypertension (Lynch *et al*. 2014) and Meta Salud Diabetes in Hispanic adults with type-2 diabetes, at high risk for cardiovascular disease (Nuño *et al*. 2023) both reported improvements in diet and physical activity behaviours, with a reduction in Hba1c.

Psychosocial outcomes were particularly responsive in interventions incorporating CBT or peer support. Skills to manage pain (STOMP) intervention demonstrated significant and sustained reductions in pain in a RCT with non-Hispanic with HIV and chronic pain (Jones *et al*. 2024), while cognitive-behavioural play therapy showed advantages over music and relaxation therapy for medication adherence in a RCT among Latino adults with asthma and panic disorder (Feldman *et al*. 2016). Feasibility and acceptability were generally high; however, attrition and incomplete intervention dose were common, particularly in pilot studies and among culturally and linguistically diverse populations. For example, Williams *et al*. (2012) found no significant improvements in medication self-efficacy or adherence following a RCT evaluating translated motivational self-management intervention in linguistically diverse (Greek, Italian, Vietnamese speakers) with chronic kidney disease, diabetes and cardiovascular disease.

#### Technology-enabled monitoring and telehealth interventions

Eighteen studies (69%) incorporated telehealth platforms, remote monitoring, or digital communication tools (telephone, smartphone apps, or videoconferencing) to extend access and continuity of care to individuals or groups. Technology was not used as a stand-alone intervention; instead, it functioned as a delivery mechanism supporting collaborative care, task shifting, or self-management strategies. Telehealth interventions were more prominent in studies published after the COVID era. For example, the STOMP intervention which started as in-person later switched to online following the COVID pandemic (Jones *et al*. 2024).

Telehealth delivery improved accessibility and continuity and were generally feasible and acceptable, particularly for individuals facing geographic or scheduling barriers. Telemonitoring paired with nurse case management produced greater BP reductions than monitoring alone, demonstrating the added value of human support (Ogedegbe *et al*. 2024). Aharonovich *et al*. (2017) RCT demonstrated that smartphone-based self-monitoring with motivational interviewing reduced substance use among urban minority adults living with HIV in the HealthCall smartphone intervention compared with motivational interviewing alone. Breslow *et al*. (2024) pilot study similarly reported high feasibility and acceptability of a group-based telehealth delivery of e-SMART/EST for older women living with HIV and experiencing past-year depression. However, Jean-Louis *et al*. (2017) tailored telephone-delivered intervention improved sleep-apnoea consultation uptake but did not improve treatment adherence in a RCT among Blacks with Metabolic Syndrome and Sleep Apnoea.

### Mapping of intervention to Ottawa Charter action areas

Across the 26 included studies, health promotion was operationalized through four Ottawa Charter action areas: developing personal skills (individual level), strengthening community actions (community level), reorienting health services (health system level), and creating supportive environments (societal level). No intervention directly addressed building healthy public policy (policy level). Interventions were also mapped to more than one action area where components aligned with multiple domains (Supplementary Files S3 and S5). All 26 studies included components aimed at developing personal skills, such as self-management education, behavioural skill-building, symptom monitoring, or motivational counselling. Creating supportive environments was evident in 23 (88%) studies, most commonly through telehealth delivery, group-based formats, home visits, or continuity across care settings. Strengthening community action was observed in 14 (54%) studies, largely those involving CHWs, *promotoras*, peer facilitators, or community-embedded group interventions. Reorienting health services was present in 21 (81%) studies, particularly within collaborative, task shifting, telehealth enabled, and integrated mental and physical health care, redistributed care roles, or extended services beyond traditional clinic encounters.

### PPIE sessions and co-production workshop

Twenty-seven individuals with MLTC or caring for family member with MLTC participated in four PPIE groups. Two groups were held with South Asians (one in-person group with women only in May 2025 and an online group with men only in July 2025). One in-person group was held with Black Africa and Caribbean women in May 2025. The last group was online and with a mix of South Asian and Black African men and women held in November 2025. In addition, 31 members of the public, community representatives and academic researchers took part in a half-day in-person dissemination and co-production workshop in November 2025.

PPIE participants expressed surprise, shock and disappointment upon learning there were no UK-based MLTC interventions identified in the scoping review that specifically target ethnic minority populations. Participants questioned where and how researchers recruit, highlighting a perceived under-representation in academic research practices. Several interrelated reasons for the under-representation of ethnic minority populations in MLTC research were identified. A prominent theme was a trust deficit, including a lingering perception of being treated as ‘guinea pigs’. This mistrust was linked to historical experiences, limited meaningful engagement with health services, and the belief that research and healthcare systems are not designed to serve ethnic minority communities.

Regarding health conditions, PPIE participants expressed dominant physical conditions diabetes and hypertension resonated with their groups, however the Black and Caribbean PPIE group felt this did not highlight conditions which feature in their communities, like cancer and sickle cell anaemia. On the other hand, the co-occurrence of physical and mental conditions resonated with the South Asian communities. Participants further added that stigma persist particularly in relation to cancer, mental health conditions and substance misuse. This was compounded by what one participant in the men-only PPIE group described as ‘cultural dishonesty’, a term they used to describe their perception that illness may sometimes go unrecognized or be concealed because of cultural norms, or limited health understanding among some ethnic minority communities in the UK. Finally, participants felt outcomes from the review were too clinical and restrictive. They emphasized the importance of outcomes beyond clinical indicators and that captured their lived experiences, legacy and generational impact, including lifestyle change, effects on family health behaviours, and improvements in health literacy across household members. In terms of recommendations, while multi-level interventions (spanning individual, community, and healthcare system levels) were considered ideal, participants felt these would be complex to implement initially. Community-level approaches were therefore recommended as a pragmatic starting point, particularly through trusted intermediaries such as CHWs.

A central theme from the co-production workshop was the importance of collaborative approaches, with emphasis on co-creation between researchers and communities. This was reflected in a cyclical model of engagement: act, share, learn and understand (Fig. 2). Community members further emphasized the need for dissemination strategies that are accessible, culturally appropriate, and community oriented.

**Figure 2:**
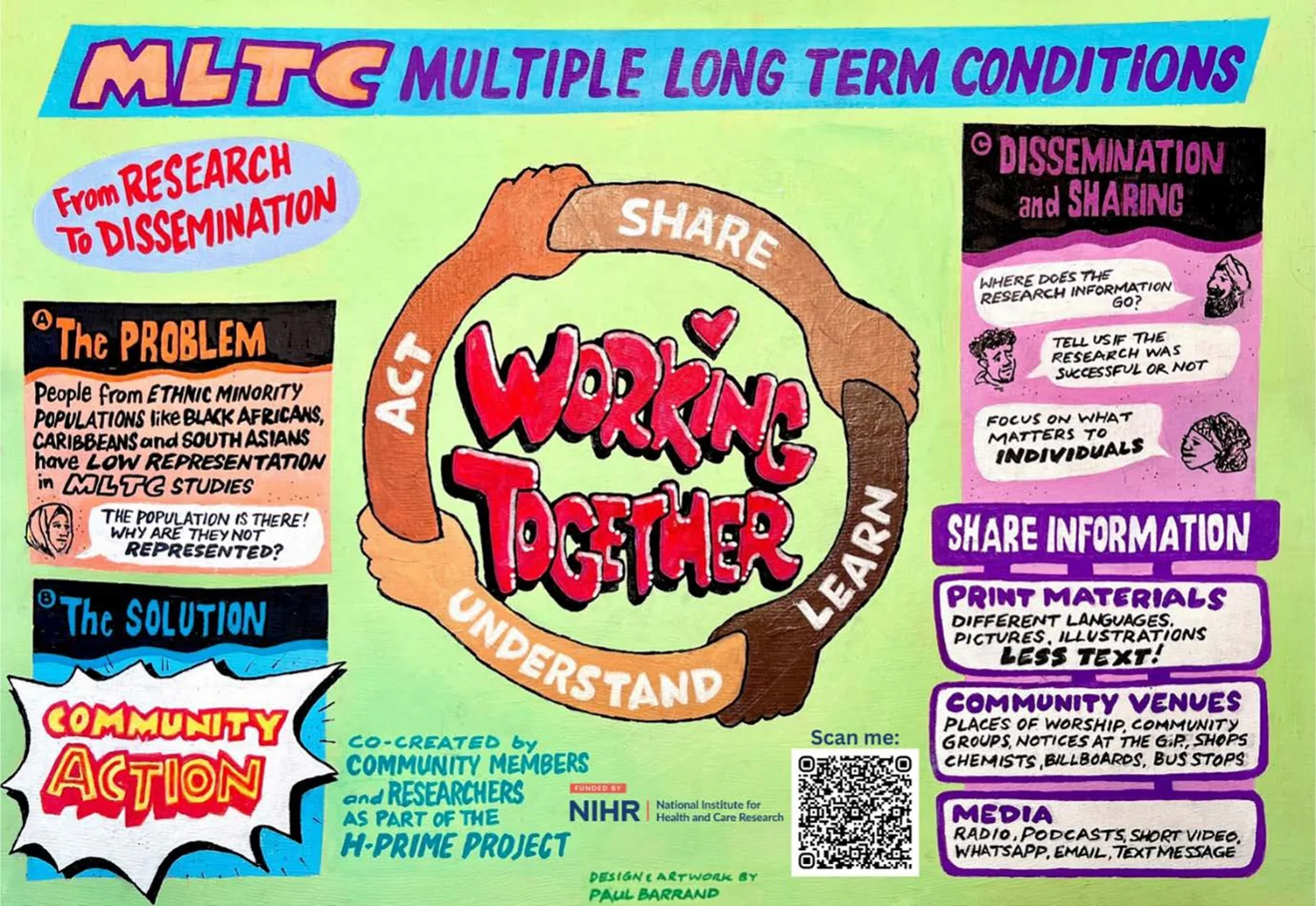
Illustration from the co-production workshop with community members. Source: Ikhile (2026) Health promotion interventions for multiple long-term conditions in ethnic minority populations (H-PRIME). University of Leicester. Figure. Available via Figshare, DOI: https://doi.org/10.25392/leicester.data.31904566.v1. Licensed under Creative Commons Attribution 4.0 International (CC BY 4.0): https://creativecommons.org/licenses/by/4.0/.

## Discussion

This scoping review mapped existing interventions for MLTC and demonstrates that, despite growing evidence on the burden of MLTC, particularly among ethnic minority populations (Hayanga *et al*. 2023, Valabhji *et al*. 2024), the evidence base remains geographically narrow. Specifically, the absence of UK-based, ethnicity-specific MLTC interventions reveals a gap between policy commitments to equity and intervention delivery. This finding is consistent with recent systematic reviews published in 2024 and 2026, which showed under-representation of ethnic minority populations in MLTC research (Kayani *et al*. 2024, Hayanga *et al*. 2026). Themes identified through PPIE, including mistrust, stigma, and cultural silence mirror well-documented barriers to research participation and sustained engagement (Shefer *et al*. 2013, Pardhan *et al*. 2025), suggesting that the limited reach of MLTC interventions among ethnic minority groups reflects systemic shortcomings in health research rather than lack of interest or capability within these communities.

Across the included studies, interventions predominantly focused on self-management education, behavioural skills, and psychosocial coping, reflecting the dominance of individual-level behaviour change paradigms in chronic disease management. While these approaches showed some improvements in proximal outcomes such as self-efficacy, their effects on clinical markers were variable. This pattern reflects a longstanding critique in health promotion and social determinants literature, which argues that individual-level skills development alone is insufficient when the social, economic, and healthcare contexts shaping individuals’ capacity to act remain unaddressed (Wilkinson and Marmot 2003, Holman *et al*. 2018). Analysed through the Ottawa Charter action area lens, the findings underscore the limitations of self-management education-centred approaches and the need for multi-level action that addresses contextual and structural determinants of MLTC burden.

Interventions extending beyond individual-level strategies, most notably collaborative and integrated care models, demonstrated the clearest and most sustained benefits, aligning with the Ottawa Charter’s call to reorient health services. Collaborative models that explicitly integrate physical and mental healthcare, particularly for common condition clusters such as diabetes and depression or hypertension and depression, were associated with improvements in depressive symptoms, functional outcomes, medication adherence, and, in some cases, cardiometabolic indicators including BP control. These findings are consistent with evidence from meta-analyses and key trials, which show that collaborative care significantly improves depression outcomes and treatment adherence, with variable effects on cardiometabolic outcomes (Katon *et al*. 2010, Wang *et al*. 2022, Cooper *et al*. 2024). For example, TEAMcare, a landmark trial in the USA, found significant improvements in both depression remission and adherence to antidepressant and diabetes medications, alongside modest improvements in HbA1c and cardiovascular risk factors (Katon *et al*. 2010). These findings reinforce the need for health systems to scale and sustain integrated, multidisciplinary models of care, moving beyond siloed approaches to better support the complex needs of populations living with MLTC.

Consistent with the wider literature, task shifting of chronic care to non-physician providers including nurses, pharmacists, and CHWs was highlighted as a feasible strategy to expand service delivery and improve access to care, particularly in the context of workforce constraints (Anwar 2025, Gong *et al*. 2025, Cassambai *et al*. 2026). However, outcomes appear contingent on how these roles are operationalized and the extent to which interventions move beyond clinical tasks to addressing social barriers. In this review, CHW-led models improved engagement as well as clinical indicators. These benefits are likely attributable to CHWs’ ability to provide trusted, culturally concordant support, and help addressing the social and practical barriers to MLTC management. In contrast, nurse-led task-shifting interventions included in this review demonstrated more variable effects on clinical outcomes. These findings suggest that the effectiveness of task shifting in MLTC management may depend less on professional substitution alone and more on the relational, contextual, and supportive work that enables individuals to navigate the complexities of living with and managing the complexity of MLTC management (Buthelezi *et al*. 2025, Yousefi Nooraie *et al*. 2025).

The prominence of telehealth across the included studies reflects a shift towards digitally enabled MLTC care, particularly in the post-COVID era (Jerjes and Harding 2024). However, telehealth primarily functioned as an enabling infrastructure rather than a stand-alone intervention, enhancing existing models such as collaborative care and task shifting. This is consistent with wider evidence that digital interventions are most effective when embedded within broader care systems rather than implemented in isolation (Kraef *et al*. 2020, Cassambai *et al*. 2026). Telehealth improved access and continuity, aligning with creating supportive environments and reorienting health services within the Ottawa Charter framework by reducing geographical and structural barriers. However, outcomes remained variable, with limited impact on sustained adherence, consistent with critiques that digital and individual-level interventions are insufficient when broader social determinants remain unaddressed (Holman *et al*. 2018, Kraef *et al*. 2020). Furthermore, the expansion of telehealth raises concerns regarding equity and digital exclusion, as access to technology and digital literacy remains uneven particularly among socioeconomically disadvantaged and ethnic minority populations (Nchafack and Ikhile 2020, Jerjes and Harding 2024).

Across all intervention types, a striking gap was the absence of explicit engagement with the Ottawa Charter domain of building healthy public policy. Responsibility for managing MLTC was predominantly largely positioned at the level of individuals and health services, with limited attention to the wider structural and policy factors. This finding is particularly significant given that the Ottawa Charter explicitly positions health promotion as a process that extends beyond healthcare and requires policy action across sectors to address the conditions that produce health inequities (Thomas *et al*. 2025). Previous studies have also highlighted the limited attention given to upstream determinants of MLTC, including housing conditions, employment, socioeconomic deprivation and neighbourhood environments that disproportionately shape MLTC risk in ethnic minority populations (Ingram *et al*. 2021, Hayanga *et al*. 2023). This downstream orientation limits the transformative potential of current interventions and risks framing MLTC as a problem of adaptation rather than one of structural inequality. Without integration of policy-level action, health promotion efforts may mitigate burden, but cannot fundamentally alter the unequal distribution of MLTC outcomes.

A key finding from the review is the limited and inconsistent use of theory and frameworks across interventions. This impedes the reproducibility and generalizability of existing interventions as theories and frameworks aid understanding of the mechanisms through which interventions operate and how change occurs (Cowdell and Dyson 2019, Skivington *et al*. 2021). There was a clear predominance of individual-level behaviour change theories, which further reflects a narrow focus on individual-level determinants. While appropriate for influencing behaviour, this orientation may inadequately capture the complex social, system and structural drivers of MLTC, particularly in ethnic minority populations. The application of ADAPT-ITT framework, the PRECEDE model, and the socio-ecological model offer a more holistic, multi-level perspective, incorporating contextual, environmental, and implementation considerations. The socio-ecological model specifically aligns with the Ottawa charter action areas and PPIE recommendation for multi-level interventions, as it reinforces that health is produced through interacting influences across individual, interpersonal, organization, community and policy levels, and that effective interventions require attention to the structural determinants of health (McLeroy *et al*. 1988, Thomas *et al*. 2025). However, limited use of such frameworks suggests that multi-level underpinnings of MLTC interventions remain underdeveloped within the current evidence base.

### Implications for research, practice, and policy

The findings from this review indicate that multicomponent interventions operating across multiple Ottawa Charter action areas have the potential to address the complexity of MLTC equitably. Individual-level education without service reorientation or environmental support is unlikely to yield sustained biomedical gains, while system-level reforms that neglect skill-building and community engagement risk overlooking lived experience and treatment burden. Effective MLTC responses therefore require integration of health promotion principles with theory-informed and multi-level frameworks linking individual agency, relational care, supportive environments, and upstream policy change. This synthesis positions health promotion not as an adjunct to MLTC care, but as a necessary foundation for addressing MLTC in ethnic minority populations.

Future research should prioritize longer follow-up periods, implementation-focused designs, and outcome measures that capture individual experiences, carers/family outcomes, care continuity and wellbeing. From a practice perspective, the evidence supports investment in multi-level programmes, complex interventions, collaborative care infrastructure, and technology-enabled but person-centred models of MLTC management. From a policy standpoint, the absence of economic evaluations limits the relevance of current evidence for commissioning; embedding cost-effectiveness analyses in future studies is essential to support scale-up (Bankolé *et al*. 2025). Stronger collaboration with multidisciplinary stakeholders is also needed to design and deliver meaningful, contextually appropriate and equity-oriented MLTC interventions for ethnic minority populations.

### Strengths and limitations

This review has several strengths. To our knowledge, it is the first to synthesize MLTC interventions from a health promotion perspective explicitly focused on ethnic minority populations, enabling analysis across individual, community, service, and policy levels. The integration of PPIE alongside the review strengthened interpretation, and contextual relevance of findings, particularly managing potential misalignment between the USA context and lived experiences of UK-based PPIE participants. Additionally, the search and screening strategies were comprehensive and systematic, covering multiple databases, with all studies independently reviewed by two researchers at the title/abstract, full-text, and data extraction stages to ensure rigour and consistency.

However, some limitations should be noted. Definitions of MLTC remain heterogeneous, and eligibility boundaries are inherently contested (Funnell *et al*. 2026), and some preventive or single condition interventions may indirectly influence MLTC outcomes but fell outside our criteria. Also, this review may not have fully captured interventions operating at all domains of the Ottawa charter action area or the full continuum of MLTC care, from prevention to treatment and long-term management. Although the search strategy included broad terms related to health promotion and MLTC management, it did not explicitly incorporate specific terminology relating to the Ottawa charter action areas or terminology relating to prevention. The exclusion of grey literature is another potential limitation as MLTC evidence may be reported in non-academic databases and organizational websites. As a result, relevant policies, strategies, or system-wide initiatives affecting the prevention and management of MLTC among ethnic minority populations may have been under-represented.

The review was restricted to OECD countries, and almost all included studies were conducted in the USA; therefore, relevant evidence from LMICs, where the burden of MLTC is also increasing, may not have been captured, potentially limiting the global generalizability of the findings. Another limitation was inconsistent reporting of race, ethnicity, and primary language across studies. As these are distinct constructs, we retained the classifications used in the original studies. However, the use of race as proxy for ethnicity in some studies may limit comparability and obscure heterogeneity within ethnic groups, and findings should therefore be interpreted with caution. Finally, the exclusion of *post hoc* or secondary analyses of trials not originally designed for MLTC may have resulted in missed insights into differential effects by ethnicity or socioeconomic position. Given the rapidly evolving nature of MLTC research, periodic updates of evidence syntheses are warranted to ensure emerging policy, practice, and research developments are adequately reflected. Particular focus should be placed on expanding the search terms to include all relevant concepts such as policy-focused terminologies where appropriate and including a range of academic databases and grey sources.

## Conclusion

This scoping review highlights a growing but uneven evidence base for interventions addressing MLTC among ethnic minority populations. Current approaches are characterized by strong emphasis on individual skill-building, supportive environments, and service-level reorientation, but limited engagement with upstream policy determinants. However, the absence of explicit policy-level action reveals a critical gap. Addressing MLTC equitably will require moving beyond downstream models towards multilevel, health promotion-informed strategies that integrate individual empowerment, community action, service re-design, and policy reform.

## Supplementary Material

daag147_Supplementary_Data

## Data Availability

The collated dataset in the current study is available from the corresponding author upon reasonable request.
