# Supplementary material for: Health promotion interventions for ethnic minority populations living with multiple long-term conditions (H-PRIME) in OECD countries: a scoping review
Source: Health Promot Int. 2026 Oct 27;41(5):daag147. doi: 10.1093/heapro/daag147 (PMC13712133; doi:10.1093/heapro/daag147)
Supplement: Supplementary file 1 [file daag147_supplementary_data.zip › Supplementary File 2_All search strategies.docx]

**Health Promotion Interventions for Ethnic Minority Populations living with Multiple Long-Term Conditions (H-PRIME) in OECD Countries: A Scoping Review**

**Search strategy concepts with details of inclusion criteria, based on the PCC (Population, Concept, Context) framework**

| Population | Individuals of any age from an ethnic minority population (as defined by the primary article) living with multiple long-term conditions (MLTC). MLTC was defined as the “The co-existence of two or more chronic conditions” (The Academy of Medical Sciences, 2018), including:   - A physical non-communicable disease of long duration, such as cardiovascular disease or cancer. - A mental health condition of long duration, such as depression or dementia. - An infectious disease of long duration, such as HIV or hepatitis C. |
| --- | --- |
| Concept | Health promotion interventions implemented to tackle MLTC. Due to the complexity and ambiguity of health promotion interventions, we will focus on interventions that embed at least one of the five health promotion action areas:   1. Developing personal skills 2. Strengthening community actions 3. Reorienting healthcare services e.g., strengthened referral, multi-professionalism, 4. Creating supportive environments 5. Building healthy public policy   Based on these action areas, interventions focusing on one or more of the following levels will be eligible:   - ***Individual level interventions*** are defined as those focused on developing personal skills to manage MLTC. This will include patient-oriented interventions as defined by the Cochrane Effective Practice and Organisation of Care (EPOC) Group Taxonomy for interventions (EPOC, 2015) e.g., self-management. - ***Community level interventions*** are defined as those focused on fostering community participation and actions to enable individual or population-based management of MLTC. Examples of community level interventions will include community or group empowerment programmes, community-wide health education campaigns, community champions/volunteers’ programmes, and faith-based programmes. - ***Healthcare level interventions*** are defined as those focused on reorienting healthcare services geared towards quality, accessible, cost-effective, timely and appropriate MLTC care. This will include healthcare professional and organizational interventions according to the EPOC taxonomy, e.g., case management, care coordination, and multidisciplinary care. - ***Societal-level interventions*** are defined as those focused on creating supportive environments by addressing the social determinants of MLTC. This will include interventions that target the conditions where people live, work and grow, e.g., interventions on the built environment or natural environment. - ***Policy-level interventions*** are defined as those focused on developing or implementing healthy public policies, strategies or initiatives to foster change in MLTC management e.g., regulatory interventions such as free primary care for MLTC prevention (Smith *et al.*, 2016).   Studies focusing on complex interventions will also be eligible as these interventions typically involve interaction of multiple levels and elements. |
| Context | Any context/setting where the intervention was delivered within an Organisation for Economic Co-operation and Development (OECD) country. **OECD Members:** [**https://www.oecd.org/en/about/members-partners.html**](https://www.oecd.org/en/about/members-partners.html)   1. Australia 2. Austria 3. Belgium 4. Canada 5. Chile 6. Colombia 7. Costa Rica 8. Czechia 9. Denmark 10. Estonia 11. Finland 12. France 13. Germany 14. Greece 15. Hungary 16. Iceland 17. Ireland 18. Israel 19. Italy 20. Japan 21. Korea 22. Latvia 23. Lithunia 24. Luxemborg 25. Mexico 26. Netherlands 27. New Zealand 28. Norway 29. Poland 30. Portugal 31. Slovak Republic 32. Slovenia 33. Spain 34. Sweden 35. Switzerland 36. Türkiye 37. United Kingdom 38. United States of America |

**References**

Effective Practice and Organisation of Care (EPOC). EPOC Taxonomy 2015. epoc.cochrane.org/epoc-taxonomy (last accessed 24 August 2023)

The Academy of Medical Sciences. Multimorbidity: a priority for global health research. The Academy of Medical Sciences: London, UK 2018

Smith SM, Wallace E, O'Dowd T *et al*. Interventions for improving outcomes in patients with multimorbidity in primary care and community settings. Cochrane Database of Systematic Reviews. 2016(3). <https://doi.org/>1002/14651858.CD006560.pub3

**Search results for all databases and Google Scholar**

**OVID Medline**

Ovid MEDLINE(R) ALL <1946 to January 24, 2025>

1	("multiple long-term conditions" or "multiple long term conditions" or MLTC).ti,ab,kf.	634

2	exp Multimorbidity/ or Comorbidity/	132880

3	(multimorbidit* or "multi-morbidit*" or "multi morbidit*" or polypharmacy or "concurrent conditions" or Comorbidit* or "co morbidit*" or "chronic conditions" or "chronic illness*" or "chronic disease*" or "chronic disorders").ti,ab,kf.	395518

4	1 or 2 or 3	473947

5	("Black, Asian and Minority Ethnic" or "Black and Minority Ethnic" or "BAME" or BME or "African continental ancestry group" or Arab or Africa* or Afro* or Asian or "Asian Continental Ancestry Group" or "South Asian" or "European Continental Ancestry Group" or Black or Caribbean or China or Chinese or Gypsy or India* or Irish or "Other White" or Pakistan* Bangladesh* or Roma or "White Other" or refugee* or "migrant*" or immigrant* or "asylum seeker*").ti,ab,kf.	1508180

6	"Ethnicity"/ or Minority Groups/ or "Race Factors"/ or "Racial Groups"/ or ethnology.fs.	264157

7	((children or communit* or faculty or men or patien* or people or person or persons or women) adj2 colo?r).mp. [mp=title, book title, abstract, original title, name of substance word, subject heading word, floating sub-heading word, keyword heading word, organism supplementary concept word, protocol supplementary concept word, rare disease supplementary concept word, unique identifier, synonyms, population supplementary concept word, anatomy supplementary concept word]	5700

8	(ethnic or ethnical or ethnically or ethnicized or ethnicities or ethnicism or ethnicities or ethnicity or ethnics or ethnic or minorities or minority or race or races or racial or racially).ti,ab,kf,kw.	410176

9	5 or 6 or 7 or 8	1878630

10	((health* or wellness) adj2 (promotion* or campaign* or program* or project* or intervention* or initiative* or audit or evaluation or evaluative)).ti,ab,kf.	179469

11	exp Early Intervention, Educational/ or exp Crisis Intervention/ or exp Internet-Based Intervention/ or exp Early Medical Intervention/ or exp Psychosocial Intervention/	16427

12	(intervention* adj2 (program* or meeting* or session* or strateg* or workshop* or visit* or education*)).mp. [mp=title, book title, abstract, original title, name of substance word, subject heading word, floating sub-heading word, keyword heading word, organism supplementary concept word, protocol supplementary concept word, rare disease supplementary concept word, unique identifier, synonyms, population supplementary concept word, anatomy supplementary concept word]	93140

13	10 or 11 or 12	275349

14	4 and 9 and 13	2783

15	limit 14 to english language	2700

16	limit 15 to dt=20250127-20250630 [January 27th, 2025 to June 30th, 2025]	182

**Cochrane Library**

ID	Search

#1	("multiple long-term conditions" or "multiple long term conditions" or MLTC):ti,ab,kw 24

#2	MeSH descriptor: [Multimorbidity] explode all trees 172

#3	MeSH descriptor: [Comorbidity] explode all trees 5344

#4	(multimorbidit* or multi-morbidit* or (multi NEXT morbidit*) or polypharmacy or (conc* NEXT conditions) or Comorbidit* or co morbidit* or (chronic NEXT conditions) or (chronic NEXT illness*) or (chronic NEXT disease*) or (chronic NEXT disorders)):ti,ab,kw 63842

#5	#1 OR #2 OR #3 OR #4 63857

#6	MeSH descriptor: [Ethnicity] explode all trees 3549

#7	MeSH descriptor: [Minority Groups] explode all trees 620

#8	MeSH descriptor: [Race Factors] explode all trees 50

#9	MeSH descriptor: [Racial Groups] explode all trees 4670

#10	MeSH descriptor: [Ethnology] explode all trees 9

#11	("Black, Asian and Minority Ethnic" or "Black and Minority Ethnic" or "BAME" or BME or "African continental ancestry group" or Arab or Africa* or Afro* or Asian or "Asian Continental Ancestry Group" or "South Asian" or "European Continental Ancestry Group" or Black or Caribbean or China or Chinese or Gypsy or India* or Irish or "Other White" or Pakistan* Bangladesh* or Roma or "White Other" or refugee* or migrant* or immigrant* or (asylum NEXT seeker*)):ti,ab,kw 119162

#12	((children or communit* or faculty or men or patien* or people or person or persons or women) NEAR/2 colo?r) 502

#13	(ethnic or ethnical or ethnically or ethnici*ed or ethnicities or ethnicism or ethnicities or ethnicity or ethnics or ethnic or minorities or minority or race or races or racial or racially):ti,ab,kw 31305

#14	#6 OR #7 OR #8 OR #9 OR #10 OR #11 OR #12 OR #13 141969

#15	((health* or wellness) NEAR/2 promotion* or campaign* or program* or project* or intervention* or initiative* or audit or evaluation or evaluative):ti,ab,kw 900583

#16	MeSH descriptor: [Early Intervention, Educational] explode all trees 718

#17	MeSH descriptor: [Crisis Intervention] explode all trees 362

#18	MeSH descriptor: [Internet-Based Intervention] explode all trees 816

#19	MeSH descriptor: [Early Medical Intervention] explode all trees 565

#20	MeSH descriptor: [Psychosocial Intervention] explode all trees 317

#21	(intervention* NEAR/2 (program* or meeting* or session* or strateg* or workshop* or visit* or education*)) 35770

#22	#15 OR #16 OR #17 OR #18 OR #19 OR #20 OR #21 900584

#23	#5 AND #14 AND #22 3502

#24	Limit #23 to English Language (NOTE: excluded 5 editorials from download) 3460

#25	Limit #23 to English Language (NOTE: excluded 5 editorials from download) with Cochrane Library publication date from Jan 2025 to Jun 2025 222

**Scopus:**

( ( TITLE-ABS-KEY ( ( "multiple long-term conditions" OR "multiple long term conditions" OR mltc ) ) OR TITLE-ABS-KEY ( ( multimorbidit* OR "multi-morbidit*" OR "multi morbidit*" OR polypharmacy OR "concurrent conditions" OR comorbidit* OR "co morbidit*" OR "chronic conditions" OR "chronic illness*" OR "chronic disease*" OR "chronic disorders" ) ) ) ) AND ( ( TITLE-ABS-KEY ( ( ethnic OR ethnical OR ethnically OR ethnici*ed OR ethnicities OR ethnicism OR ethnicities OR ethnicity OR ethnics OR ethnic OR minorities OR minority OR race OR races OR racial OR racially ) ) OR TITLE-ABS-KEY ( ( "Black, Asian and Minority Ethnic" OR "Black and Minority Ethnic" OR "BAME" OR bme OR "African continental ancestry group" OR arab OR africa* OR afro* OR asian OR "Asian Continental Ancestry Group" OR "South Asian" OR "European Continental Ancestry Group" OR black OR caribbean OR china OR chinese OR gypsy OR india* OR irish OR "Other White" OR pakistan* AND bangladesh* OR roma OR "White Other" OR refugee* OR "migrant*" OR immigrant* OR "asylum seeker*" ) ) ) ) AND ( ( TITLE-ABS-KEY ( ( health* OR wellness ) W/2 ( promotion* OR campaign* OR program* OR project* OR intervention* OR initiative* OR audit OR evaluation OR evaluative ) ) OR TITLE-ABS-KEY ( intervention* W/2 ( program* OR meeting* OR session* OR strateg* OR workshop* OR visit* OR education* ) ) ) ) AND ( LIMIT-TO ( LANGUAGE , "English" ) ) 2731 (27/01/25) 145 (30/06/25)

**PsychINFO**

| **#** | **Query** | **Limiters/Expanders** | **Results** |
| --- | --- | --- | --- |
| S17 | S8 AND S11 AND S14 | Limiters - English language, 01/2025 to 06/2025) Expanders - Apply equivalent subjects Search modes - Proximity | 18 |
| S16 | S8 AND S11 AND S14 | Limiters - English language Expanders - Apply equivalent subjects Search modes - Proximity | 795 |
| S15 | S8 AND S11 AND S14 | Expanders - Apply equivalent subjects Search modes - Proximity | 795 |
| S14 | S12 OR S13 | Expanders - Apply equivalent subjects Search modes - Proximity | 112,565 |
| S13 | TI ( ( intervention* W2 ( program* OR meeting* OR session* OR strateg* OR workshop* OR visit* OR education* ) ) ) OR AB ( ( intervention* W2 ( program* OR meeting* OR session* OR strateg* OR workshop* OR visit* OR education* ) ) ) OR KW ( ( intervention* W2 ( program* OR meeting* OR session* OR strateg* OR workshop* OR visit* OR education* ) ) ) | Expanders - Apply equivalent subjects Search modes - Proximity | 46,820 |
| S12 | TI ( ( ( health* OR wellness ) W2 ( promotion* OR campaign* OR program* OR project* OR intervention* OR initiative* OR audit OR evaluation OR evaluative ) ) ) OR AB ( ( ( health* OR wellness ) W2 ( promotion* OR campaign* OR program* OR project* OR intervention* OR initiative* OR audit OR evaluation OR evaluative ) ) ) OR KW ( ( ( health* OR wellness ) W2 ( promotion* OR campaign* OR program* OR project* OR intervention* OR initiative* OR audit OR evaluation OR evaluative ) ) ) | Expanders - Apply equivalent subjects Search modes - Proximity | 68,634 |
| S11 | S9 OR S10 | Expanders - Apply equivalent subjects Search modes - Proximity | 527,513 |
| S10 | TI ( "Black, Asian and Minority Ethnic" OR "Black and Minority Ethnic" OR "BAME" OR bme OR "African continental ancestry group" OR arab OR africa* OR afro* OR asian OR "Asian Continental Ancestry Group" OR "South Asian" OR "European Continental Ancestry Group" OR black OR caribbean OR china OR chinese OR gypsy OR india* OR irish OR "Other White" OR pakistan* AND bangladesh* OR roma OR "White Other" OR refugee* OR "migrant*" OR immigrant* OR "asylum seeker*" ) OR AB ( "Black, Asian and Minority Ethnic" OR "Black and Minority Ethnic" OR "BAME" OR bme OR "African continental ancestry group" OR arab OR africa* OR afro* OR asian OR "Asian Continental Ancestry Group" OR "South Asian" OR "European Continental Ancestry Group" OR black OR caribbean OR china OR chinese OR gypsy OR india* OR irish OR "Other White" OR pakistan* AND bangladesh* OR roma OR "White Other" OR refugee* OR "migrant*" OR immigrant* OR "asylum seeker*" ) OR KW ( "Black, Asian and Minority Ethnic" OR "Black and Minority Ethnic" OR "BAME" OR bme OR "African continental ancestry group" OR arab OR africa* OR afro* OR asian OR "Asian Continental Ancestry Group" OR "South Asian" OR "European Continental Ancestry Group" OR black OR caribbean OR china OR chinese OR gypsy OR india* OR irish OR "Other White" OR pakistan* AND bangladesh* OR roma OR "White Other" OR refugee* OR "migrant*" OR immigrant* OR "asylum seeker*" ) | Expanders - Apply equivalent subjects Search modes - Proximity | 380,595 |
| S9 | TI ( ethnic OR ethnical OR ethnically OR ethnici*ed OR ethnicities OR ethnicism OR ethnicities OR ethnicity OR ethnics OR ethnic OR minorities OR minority OR race OR races OR racial OR racially ) OR AB ( ethnic OR ethnical OR ethnically OR ethnici*ed OR ethnicities OR ethnicism OR ethnicities OR ethnicity OR ethnics OR ethnic OR minorities OR minority OR race OR races OR racial OR racially ) OR KW ( ethnic OR ethnical OR ethnically OR ethnici*ed OR ethnicities OR ethnicism OR ethnicities OR ethnicity OR ethnics OR ethnic OR minorities OR minority OR race OR races OR racial OR racially ) | Expanders - Apply equivalent subjects Search modes - Proximity | 240,817 |
| S8 | S6 OR S7 | Expanders - Apply equivalent subjects Search modes - Proximity | 84,490 |
| S7 | TI ( multimorbidit* OR "multi-morbidit*" OR "multi morbidit*" OR polypharmacy OR "concurrent conditions" OR comorbidit* OR "co morbidit*" OR "chronic conditions" OR "chronic illness*" OR "chronic disease*" OR "chronic disorders" ) OR AB ( multimorbidit* OR "multi-morbidit*" OR "multi morbidit*" OR polypharmacy OR "concurrent conditions" OR comorbidit* OR "co morbidit*" OR "chronic conditions" OR "chronic illness*" OR "chronic disease*" OR "chronic disorders" ) OR KW ( multimorbidit* OR "multi-morbidit*" OR "multi morbidit*" OR polypharmacy OR "concurrent conditions" OR comorbidit* OR "co morbidit*" OR "chronic conditions" OR "chronic illness*" OR "chronic disease*" OR "chronic disorders" ) | Limiters - English language Expanders - Apply equivalent subjects Search modes - Proximity | 84,476 |
| S6 | TI ( "multiple long-term conditions" OR "multiple long term conditions" OR mltc ) OR AB ( "multiple long-term conditions" OR "multiple long term conditions" OR mltc ) OR KW ( "multiple long-term conditions" OR "multiple long term conditions" OR mltc ) | Limiters - English language Expanders - Apply equivalent subjects Search modes - Proximity | 37 |
| S5 | S1 AND S2 AND S3 | Limiters - English language Expanders - Apply equivalent subjects Search modes - Proximity | 2,390 |
| S4 | S1 AND S2 AND S3 | Expanders - Apply equivalent subjects Search modes - Proximity | 2,404 |
| S3 | ( ( health* OR wellness ) W2 ( promotion* OR campaign* OR program* OR project* OR intervention* OR initiative* OR audit OR evaluation OR evaluative ) ) OR ( intervention* W2 ( program* OR meeting* OR session* OR strateg* OR workshop* OR visit* OR education* ) ) | Expanders - Apply equivalent subjects Search modes - Proximity | 170,513 |
| S2 | ( ethnic OR ethnical OR ethnically OR ethnici*ed OR ethnicities OR ethnicism OR ethnicities OR ethnicity OR ethnics OR ethnic OR minorities OR minority OR race OR races OR racial OR racially ) OR ( "Black, Asian and Minority Ethnic" OR "Black and Minority Ethnic" OR "BAME" OR bme OR "African continental ancestry group" OR arab OR africa* OR afro* OR asian OR "Asian Continental Ancestry Group" OR "South Asian" OR "European Continental Ancestry Group" OR black OR caribbean OR china OR chinese OR gypsy OR india* OR irish OR "Other White" OR pakistan* AND bangladesh* OR roma OR "White Other" OR refugee* OR "migrant*" OR immigrant* OR "asylum seeker*" ) | Expanders - Apply equivalent subjects Search modes - Proximity | 769,837 |
| S1 | ( "multiple long-term conditions" OR "multiple long term conditions" OR mltc ) OR ( multimorbidit* OR "multi-morbidit*" OR "multi morbidit*" OR polypharmacy OR "concurrent conditions" OR comorbidit* OR "co morbidit*" OR "chronic conditions" OR "chronic illness*" OR "chronic disease*" OR "chronic disorders" ) | Expanders - Apply equivalent subjects Search modes - Proximity | 147,792 |

**CINAHL**

| **#** | **Query** | **Limiters/Expanders** | **Results** |
| --- | --- | --- | --- |
|  |  | Expanders - Apply equivalent subjects Narrow by Language: - english Search modes – Proximity – 01/2025 to 06/2025 | 70 |
| S11 | S3 AND S6 AND S9 | Expanders - Apply equivalent subjects Narrow by Language: - english Search modes - Proximity | 984 |
| S10 | S3 AND S6 AND S9 | Expanders - Apply equivalent subjects Search modes - Proximity | 996 |
| S9 | S7 OR S8 | Expanders - Apply equivalent subjects Search modes - Proximity | 121,523 |
| S8 | TI ( (intervention* W2 ( program* OR meeting* OR session* OR strateg* OR workshop* OR visit* OR education* ) ) ) OR AB ( (intervention* W2 ( program* OR meeting* OR session* OR strateg* OR workshop* OR visit* OR education* ) ) ) | Expanders - Apply equivalent subjects Search modes - Proximity | 33,065 |
| S7 | TI ( ( ( health* OR wellness ) W2 ( promotion* OR campaign* OR program* OR project* OR intervention* OR initiative* OR audit OR evaluation OR evaluative ) ) ) OR AB ( ( ( health* OR wellness ) W2 ( promotion* OR campaign* OR program* OR project* OR intervention* OR initiative* OR audit OR evaluation OR evaluative ) ) ) | Expanders - Apply equivalent subjects Search modes - Proximity | 91,432 |
| S6 | S4 OR S5 | Expanders - Apply equivalent subjects Search modes - Proximity | 471,806 |
| S5 | TI ( "Black, Asian and Minority Ethnic" OR "Black and Minority Ethnic" OR "BAME" OR bme OR "African continental ancestry group" OR arab OR africa* OR afro* OR asian OR "Asian Continental Ancestry Group" OR "South Asian" OR "European Continental Ancestry Group" OR black OR caribbean OR china OR chinese OR gypsy OR india* OR irish OR "Other White" OR pakistan* AND bangladesh* OR roma OR "White Other" OR refugee* OR "migrant*" OR immigrant* OR "asylum seeker*" ) OR AB ( "Black, Asian and Minority Ethnic" OR "Black and Minority Ethnic" OR "BAME" OR bme OR "African continental ancestry group" OR arab OR africa* OR afro* OR asian OR "Asian Continental Ancestry Group" OR "South Asian" OR "European Continental Ancestry Group" OR black OR caribbean OR china OR chinese OR gypsy OR india* OR irish OR "Other White" OR pakistan* AND bangladesh* OR roma OR "White Other" OR refugee* OR "migrant*" OR immigrant* OR "asylum seeker*" ) | Expanders - Apply equivalent subjects Search modes - Proximity | 370,516 |
| S4 | TI ( ethnic OR ethnical OR ethnically OR ethnici*ed OR ethnicities OR ethnicism OR ethnicities OR ethnicity OR ethnics OR ethnic OR minorities OR minority OR race OR races OR racial OR racially ) OR AB ( ethnic OR ethnical OR ethnically OR ethnici*ed OR ethnicities OR ethnicism OR ethnicities OR ethnicity OR ethnics OR ethnic OR minorities OR minority OR race OR races OR racial OR racially ) | Expanders - Apply equivalent subjects Search modes - Proximity | 158,001 |
| S3 | S1 OR S2 | Expanders - Apply equivalent subjects Search modes - Proximity | 133,356 |
| S2 | TI ( ( multimorbidit* OR "multi-morbidit*" OR "multi morbidit*" OR polypharmacy OR "concurrent conditions" OR comorbidit* OR "co morbidit*" OR "chronic conditions" OR "chronic illness*" OR "chronic disease*" OR "chronic disorders" ) OR AB ( ( multimorbidit* OR "multi-morbidit*" OR "multi morbidit*" OR polypharmacy OR "concurrent conditions" OR comorbidit* OR "co morbidit*" OR "chronic conditions" OR "chronic illness*" OR "chronic disease*" OR "chronic disorders" ) | Expanders - Apply equivalent subjects Search modes - Proximity | 133,257 |
| S1 | TI ( "multiple long-term conditions" OR "multiple long term conditions" OR mltc ) OR AB ( "multiple long-term conditions" OR "multiple long term conditions" OR mltc ) | Expanders - Apply equivalent subjects Search modes - Proximity | 147 |

**Google Scholar Search**

("multiple long-term conditions" or "multiple long term conditions" or MLTC) AND Intervention AND Ethnic 129
