# Supplementary material for: Health promotion interventions for ethnic minority populations living with multiple long-term conditions (H-PRIME) in OECD countries: a scoping review
Source: Health Promot Int. 2026 Oct 27;41(5):daag147. doi: 10.1093/heapro/daag147 (PMC13712133; doi:10.1093/heapro/daag147)
Supplement: Supplementary file 1 [file daag147_supplementary_data.zip › Supplementary File 3_Operationalisation of Ottawa Charter Area.docx]

**Health Promotion Interventions for Ethnic Minority Populations living with Multiple Long-Term Conditions (H-PRIME) in OECD Countries: A Scoping Review**

**Mapping Criteria for Ottawa Charter Action Areas**

| **Ottawa Charter Action Area** | **Definition** | **Coding criteria applied in this review** | **Studies Mapped to this Action Area** |
| --- | --- | --- | --- |
| **Building Healthy Public Policy** | Combining diverse but complementary approaches such as  Legislative or regulatory changes in order to improve MLTC outcomes. Focus is on policies that contribute to safer and healthier environment and foster greater equity (Ottawa Charter for Health Promotion 1986; Smith et al. 2016). | - Policy changes relating to health, housing, education, social care etc - Fiscal reform - Regulatory changes - Taxation on unhealthy products - Interventions explicitly targeting upstream/structural determinants | N= 0 |
| **Creating Supportive Environments** | Creating social, cultural, economic and physical environments that support health and make healthier choices easier. Involves community or organisational changes and interventions that provide services, resources, or support to individuals, helping to create physical, social, or cultural environments that are more supportive of MLTC management ( Ottawa Charter for Health Promotion 1986; Fry and Zask 2017). | - Home-based support or monitoring; - Family involvement and support; - Social support mechanisms; - Reduction of barriers to participation or healthcare access (through technology, interpreters etc); - Culturally and linguistically tailored programmes; - Use of visual aids and accessible language; - Community resource referral and linkage; - Provision of resources to facilitate self-management/behaviour change; - Provision of transportation or practical support services. | N= 23 (Aharonovich et al. 2017; Breslow et al. 2024; Diamantidis et al. 2018; Dunbar et al. 2015; Eakin et al. 2007; Ell et al. 2010; Ell et al. 2017; Feldman et al. 2016; Gallo et al. 2023; Gardiner et al. 2019; Jean-Louis et al. 2017; Jones et al. 2024; Kangovi et al. 2017; Lynch et al. 2014; Metsch et al. 2016; Metsch et al. 2021; Nuño et al. 2023; Ogedegbe et al. 2024; Rimmer et al. 2002; Shah et al. 2024; Tobe et al. 2006; Towfighi et al. 2021; Williams et al. 2012) |
| **Strengthening Community Action** | Enabling communities to identify priorities, make decisions, plan strategies and implement actions to improve health through participation, empowerment and community ownership. It involves offering opportunities or resources for collective action ( Ottawa Charter for Health Promotion; Fry and Zask 2017). | - Community health workers or Promotoras; - Peer educators or peer mentors; - Community mentors; - Community-led intervention delivery; - Participatory programmes; - Involvement of community representatives in intervention design and/or delivery; - Empowering participants to access relevant community support/resources; - Community-based programmes. | N= 14 (Breslow et al. 2024; Dunbar et al. 2015; Eakin et al. 2007; Ell et al. 2017; Gallo et al. 2023; Jean-Louis et al. 2017; Jones et al. 2024; Kangovi et al. 2017; Lynch et al. 2014; Metsch et al. 2016; Metsch et al. 2021; Nuño et al. 2023; Shah et al. 2024; Towfighi et al. 2021) |
| **Developing Personal Skills** | Supporting personal and social development by providing information, education for health and enhancing life skills that increase control over health ( Ottawa Charter for Health Promotion 1986). | - Health education; - Self-management training; - Behaviour change interventions; - Motivational interviewing; - Lifestyle counselling; - Goal setting and action planning; - Problem solving; - Self-monitoring; - Lifestyle modification; - Medication adherence support. | N= 26 (Aharonovich et al. 2017; Alexopoulos et al. 2024; Bogner and de Vries 2008; Breslow et al. 2024; Diamantidis et al. 2018; Dunbar et al. 2015; Eakin et al. 2007; Ell et al. 2010; Ell et al. 2017; Feldman et al. 2016; Gallo et al. 2023; Gardiner et al. 2019; Jean-Louis et al. 2017; Jones et al. 2024; Kangovi et al. 2017; Lynch et al. 2014; Metsch et al. 2016; Metsch et al. 2021; Nuño et al. 2023; Ogedegbe et al. 2024; Rimmer et al. 2002; Shah et al. 2024; Tobe et al. 2006; Towfighi et al. 2021; Williams et al. 2012; Zuñiga et al. 2019) |
| **Reorienting Health Services** | Refers to changes in the organisation and delivery of MLTC care that enhance person-centred approaches, multidisciplinary teamwork, care coordination, and integrated service provision. It also encompasses initiatives aimed at developing the knowledge, skills, and competencies of professionals involved in MLTC management. (Ottawa Charter for Health Promotion 1986; Smith et al. 2016). | - Integrated care models; - Collaborative care approaches; - Telehealth or telemonitoring; - Task-shifting to community health workers; - Nurse-led care; - Pharmacist-led care; - Multidisciplinary teams; - Case management; - Health services referral; - Medication review; - Patient activation and empowerment - Care facilitation or care coordination; | N= 21 (Aharonovich et al. 2017; Alexopoulos et al. 2024; Bogner and de Vries 2008; Breslow et al. 2024; Diamantidis et al. 2018; Dunbar et al. 2015; Ell et al. 2010; Ell et al. 2017; Feldman et al. 2016; Gallo et al. 2023; Gardiner et al. 2019; Kangovi et al. 2017; Metsch et al. 2016; Metsch et al. 2021; Nuño et al. 2023; Ogedegbe et al. 2024; Shah et al. 2024; Tobe et al. 2006; Towfighi et al. 2021; Williams et al. 2012; Zuñiga et al. 2019) |

**References**

Aharonovich, Efrat, et al. (2017), 'HealthCall delivered via smartphone to reduce co-occurring drug and alcohol use in HIV-infected adults: a randomized pilot trial', *Journal of Substance Abuse Treatment,* 83, 15-26.

Alexopoulos, Anastasia-Stefania, et al. (2024), 'Intervening on Metabolic Dysfunction-Associated Steatotic Liver Disease in Latino/a and Black Patients with Diabetes: A Feasibility Pilot', *Diabetes Therapy,* 15 (11), 2417-2

Bogner, Hillary R and de Vries, Heather F (2008), 'Integration of depression and hypertension treatment: a pilot, randomized controlled trial', *The Annals of Family Medicine,* 6 (4), 295-301.

Breslow, Aaron S, et al. (2024), 'Lessons learned from a community-led, pilot teletherapy group for older women living with depression and HIV', *AIDS and Behavior,* 28 (12), 4079-93.

Diamantidis, Clarissa J, et al. (2018), 'Simultaneous Risk Factor Control Using Telehealth to slOw Progression of Diabetic Kidney Disease (STOP-DKD) study: protocol and baseline characteristics of a randomized controlled trial', *Contemporary clinical trials,* 69, 28-39.

Dunbar, Sandra B, et al. (2015), 'Randomized clinical trial of an integrated self-care intervention for persons with heart failure and diabetes: quality of life and physical functioning outcomes', *Journal of cardiac failure,* 21 (9), 719-29.

Eakin, Elizabeth G, et al. (2007), 'Resources for health: a primary-care-based diet and physical activity intervention targeting urban Latinos with multiple chronic conditions', *Health Psychology,* 26 (4), 392.

Ell, Kathleen, et al. (2017), 'Promotora assisted depression and self-care management among predominantly Latinos with concurrent chronic illness: safety net care system clinical trial results', *Contemporary clinical trials,* 61, 1-9.

Ell, Kathleen, et al. (2010), 'Collaborative care management of major depression among low-income, predominantly Hispanic subjects with diabetes: a randomized controlled trial', *Diabetes care,* 33 (4), 706-13.

Feldman, Jonathan M, et al. (2016), 'Psychological treatment of comorbid asthma and panic disorder in Latino adults: Results from a randomized controlled trial', *Behaviour research and therapy,* 87, 142-54.

Fry, Denise and Zask, Avigdor (2017), 'Applying the Ottawa Charter to inform health promotion programme design', *Health Promotion International,* 32 (5), 901-12.

Gallo, Linda C, et al. (2023), 'Mi Puente (My Bridge) care transitions program for Hispanic/Latino adults with multimorbidity: results of a randomized controlled trial', *Journal of general internal medicine,* 38 (9), 2098-106.

Gardiner, Paula, et al. (2019), 'Effectiveness of integrative medicine group visits in chronic pain and depressive symptoms: A randomized controlled trial', *PloS one,* 14 (12), e0225540.

Jean-Louis, Girardin, et al. (2017), 'Tailored behavioral intervention among blacks with metabolic syndrome and sleep apnea: results of the MetSO trial', *Sleep,* 40 (1), zsw008.

Jones, Katie Fitzgerald, et al. (2024), 'Efficacy of a pain self-management intervention tailored to people with HIV: a randomized clinical trial', *JAMA internal medicine,* 184 (9), 1074-82.

Kangovi, Shreya, et al. (2017), 'Community health worker support for disadvantaged patients with multiple chronic diseases: a randomized clinical trial', *American journal of public health,* 107 (10), 1660-67.

Lynch, Elizabeth B, et al. (2014), 'A self-management intervention for African Americans with comorbid diabetes and hypertension: a pilot randomized controlled trial', *Preventing chronic disease,* 11, E90.

Metsch, Lisa R, et al. (2016), 'Effect of patient navigation with or without financial incentives on viral suppression among hospitalized patients with HIV infection and substance use: a randomized clinical trial', *Jama,* 316 (2), 156-70.

Metsch, Lisa R, et al. (2021), 'Care facilitation advances movement along the hepatitis C care continuum for persons with human immunodeficiency virus, hepatitis C, and substance use: a randomized clinical trial (CTN-0064)', *Open forum infectious diseases* (8: Oxford University Press US), ofab334.

Nuño, Tomas, et al. (2023), 'Feasibility and outcomes of Meta Salud diabetes behavioral health intervention: A pilot study of a community health worker-administered educational intervention to prevent cardiovascular disease and its complications among hispanic patients with type-2 diabetes', *International Journal of Environmental Research and Public Health,* 20 (21), 6968.

Ogedegbe, Gbenga, et al. (2024), 'Home blood pressure telemonitoring and nurse case management in Black and Hispanic patients with stroke: a randomized clinical trial', *JAMA,* 332 (1), 41-50.

OTTAWA CHARTER FOR HEALTH PROMOTION (1986), *Health Promotion International,* 1 (4), 405-05.

Rimmer, James H, et al. (2002), 'Feasibility of a health promotion intervention for a group of predominantly African American women with type 2 diabetes', *The Diabetes Educator,* 28 (4), 571-80.

Shah, Megha K, et al. (2024), 'A Culturally Adapted, Telehealth, Community Health Worker Intervention on Blood Pressure Control among South Asian Immigrants with Type II Diabetes: Results from the DREAM Atlanta Intervention: Shah et al', *Journal of General Internal Medicine,* 39 (4), 529-39.

Smith, Susan M, et al. (2016), 'Interventions for improving outcomes in patients with multimorbidity in primary care and community settings', *Cochrane Database of Systematic Reviews,* (3).

Tobe, Sheldon W, et al. (2006), 'Effect of nurse-directed hypertension treatment among First Nations people with existing hypertension and diabetes mellitus: the Diabetes Risk Evaluation and Microalbuminuria (DREAM 3) randomized controlled trial', *Cmaj,* 174 (9), 1267-71.

Towfighi, Amytis, et al. (2021), 'Effect of a coordinated community and chronic care model team intervention vs usual care on systolic blood pressure in patients with stroke or transient ischemic attack: the SUCCEED randomized clinical trial', *JAMA network open,* 4 (2), e2036227.

Williams, Allison, et al. (2012), 'Working with CALD groups: testing the feasibility of an intervention to improve medication self-management in people with kidney disease, diabetes, and cardiovascular disease'.

Zuñiga, Julie, et al. (2019), 'Pilot test of an educational intervention to improve self-management of diabetes in persons living with HIV', *Pilot and Feasibility Studies,* 5 (1), 111.
