# Supplementary material for: Health promotion interventions for ethnic minority populations living with multiple long-term conditions (H-PRIME) in OECD countries: a scoping review
Source: Health Promot Int. 2026 Oct 27;41(5):daag147. doi: 10.1093/heapro/daag147 (PMC13712133; doi:10.1093/heapro/daag147)
Supplement: Supplementary file 1 [file daag147_supplementary_data.zip › Supplementary File 4. Word cloud of conditions targeted in included studies.docx]

**Health Promotion Interventions for Ethnic Minority Populations living with Multiple Long-Term Conditions (H-PRIME) in OECD Countries: A Scoping Review**

**Supplementary File 4. Word cloud of conditions targeted in included studies**

Alt text: Word cloud showing the health conditions targeted in the included studies. The size of each word indicates the frequency of condition inclusion in the studies. Diabetes, hypertension, and HIV are the most prominent conditions, indicating they were the most frequently targeted across the included studies.
