# Supplementary material for: Health promotion interventions for ethnic minority populations living with multiple long-term conditions (H-PRIME) in OECD countries: a scoping review
Source: Health Promot Int. 2026 Oct 27;41(5):daag147. doi: 10.1093/heapro/daag147 (PMC13712133; doi:10.1093/heapro/daag147)
Supplement: Supplementary file 1 [file daag147_supplementary_data.zip › Supplementary File 5_Details of interventions.docx]

**Health Promotion Interventions for Ethnic Minority Populations living with Multiple Long-Term Conditions (H-PRIME) in OECD Countries: A Scoping Review**

**Details of interventions from included studies**

Alt text: Table presenting intervention characteristics across the included studies and intervention categories from the synthesis. It includes study identifier, intervention name and description, category, Ottawa charter action area, theory/framework, delivery mode, duration, frequency, provider, setting, adaptation and outcome measures.

| Study | Participants | Intervention name + description | Category | Ottawa Charter Action Area | Theory/  Framework | Delivery mode | Provider | Setting | Dose/  Frequency | Adaptation | Outcome measures |
| --- | --- | --- | --- | --- | --- | --- | --- | --- | --- | --- | --- |
| Aharonovich et al., 2017 | Urban minority adults living with HIV with co-occurring substance/drug use (N= 47) | **HealthCall Smartphone** A smartphone‑based intervention that supports self‑monitoring of substance use and HIV‑related behaviours, combined with motivational interviewing feedback sessions  Two arms: 1. Motivational interviewing 2. Motivational interviewing + HealthCall-Smartphone | Telehealth + self-management | Developing personal skills, reorienting health services, creating supportive environment | None reported | Smartphone self-monitoring, in-person provider feedback | Trained MI counsellor | HIV clinics and community HIV health programmes | Daily self-monitoring for 30 days. Booster sessions at 30 and 60-day visits | Smartphone technology adapted from HealthCall- interactive voice response Personalised feedback based on participant‑reported substance use data collected through the HealthCall app | **Feasibility outcomes** 1) Engagement and satisfaction 2) Intervention implementation and retention 3) Feasibility concerns 4) Additional qualitative data **Efficacy outcomes** 1) Reduction in drug use 2) Reduction in alcohol use 3) total number of drinking days 4) mean number of standard drinks per day |
| Alexopoulos et al., 2024 | Latino/a and Black patients with type 2 diabetes and metabolic dysfunction‑associated steatotic liver disease (MASLD) (N= 10) | **Evidence-based management (EBM) of MASLD** Single-arm telehealth intervention comprising: (a) MASLD education; (b) diet/lifestyle counselling; (c) T2D medication adjustment (i.e., to promote liver health) and (d) ordering of clinically indicated tests and referrals | Collaborative/integrated care + Telehealth | Developing personal skills, reorienting health services | None reported | Telehealth (via zoom or telephone) | Board-certified endocrinologist | Virtual | Three virtual visits over three months | None reported | **Feasibility** 1) Recruitment rates 2) Retention rates  3) Visit completion rates **Acceptability** Treatment Acceptability and Preferences (TAP) measure **Psychosocial** 1) SEMCD self-efficacy for managing chronic diseases scale 2) SF-12 12-item short form quality of life survey **Others** 1) Type 2 diabetes medication adjustment rates 2) Ordering of clinically indicated tests/referrals rates |
| Bogner & de Vries, 2008 | Older adults (50 - 80 years) prescribed pharmacotherapy for depression and hypertension (N= 64) | **Integrated care intervention** Integrated depression and hypertension treatment incorporating coordinated management of antidepressant and antihypertensive medications, offered as a supplement to primary care treatment.  The key components of the intervention were (1) providing the patient with an individualised program that is congruent with patients’ social and cultural context; and (2) integrating depression treatment with hypertension management. | Collaboration/integrated care + Telehealth | Developing personal skills, reorienting health services | None reported | In‑person primary care visits and telephone-monitoring | Master’s level research coordinator trained as integrated care manager | Primary care | Baseline, 2 weeks, 4 weeks and 6 weeks | Intervention conceptual framework adapted from Cooper et al. 2003 | 1) Depression severity (CES‑D) 2) Blood pressure using the BpTRU 3) Medication adherence: Adherence to antidepressant and antihypertensive medications measured using electronic monitoring data obtained from Medication Event Monitoring System caps |
| Breslow et al., 2024 | Women, 55 years and older living with HIV and experiencing past year depressive symptoms (N= 8) | **Electronic Stress Management and Relaxation Training/Expressive Supportive Therapy (e-SMART/EST)** Community‑led, group‑based teletherapy intervention adapted for older women with HIV, providing stress management, relaxation training, and expressive supportive therapy via online teletherapy group | Telehealth + self-management | Developing personal skills, reorienting health services, creating supportive environment, strengthening community action | ADAPT‑ITT framework | Zoom | Co-facilitated by a Licensed Psychologist and a credentialed Peer Counsellor | Virtual | 10 sessions: pre-group survey, 8 weekly group sessions, post group survey | Culturally and contextually tailored using ADAPT‑ITT | 1) Levels of engagement Fidelity **Feasibility, Acceptability, and Appropriateness** 1) Feasibility of Intervention Measure (FIM) 2) Acceptability of Intervention Measure (AIM)  3) Intervention Appropriateness Measure (IAM) **Depression and HIV-related Quality of life** 1) 21-item Beck Depression Inventory (BDI) 2) 12-item, condensed version of the Medical Outcomes Study-HIV Health Survey (MOS-HIV SF-12) |
| Diamantidis et al., 2018 | Adults with diabetes and uncontrolled hypertension and evidence of kidney dysfunction (N= 281) | **The Simultaneous Risk Factor Control Using Telehealth to slOw Progression of Diabetic Kidney Disease (STOP-DKD)** Multifactorial telehealth behavioural and medication‑management intervention including pharmacist‑led education modules, home blood pressure monitoring, and electronic medication management recommendations to primary care clinicians. Two arms: educational and multifactorial intervention arm. | Collaborative/integrated care + telehealth | Developing personal skills, reorienting health services, creating supportive environment | None reported | Telephone education, home blood pressure monitoring, electronic education | Clinical pharmacist | Virtual | Up to 36 monthly telephone modules; ongoing home BP monitoring | None reported | **Primary outcome**  1) Change in estimated glomerular filtration rate (eGFR) at three years **Secondary outcomes**  1) Changes in blood pressure, blood glucose, haemoglobin A1C, and urinary albumin excretion **Others**  1) psychological barriers to optimal DKD care 2) DKD and risk factor awareness and knowledge 3) behaviours such as physical activity, diet, sleep, tobacco and alcohol use,  4) sociodemographic barriers to optimal DKD care 5) Quality of life 6) Household chaos 7) Perceived self-efficacy 8) Perceived diabetes self-management 9) Social support and barriers to medication treatment. **Additional biomedical assessments** 1) Ankle brachial index 2) Retinopathy screening 3) Biorepository specimen collection 4) Ambulatory blood pressure monitoring |
| Dunbar et al., 2015 | Adults with heart failure and diabetes mellitus (N= 134) | **Integrated heart failure-diabetes mellitus self-care** Education and counselling on combined heart failure and diabetes self-care (diet, medications, self-monitoring, symptoms, and physical activity) with follow-up home visit and telephone counselling. Family members were encouraged to attend. | Collaborative/Integrated care + Telehealth | Developing personal skills, reorienting health services, creating supportive environment, strengthening community action | None reported | Hybrid: in‑person (home visit) plus telephone follow‑up | Trained research nurse | Community‑based / outpatient | Baseline, immediately after enrolment, and after 48–72 hours, 7 days, 14 days, 1 and 2 months, and 4.5 months after study entry | None reported | 1) Heart failure quality of life: Minnesota Living with Heart Failure Questionnaire (MLHFQ) 2) DM‑specific quality of life: Audit of Diabetes-Dependent Quality of Life (ADDQOL) 3) Overall quality of life: EuroQol 5-Dimension (EQ-5D) 4) Physical functioning (6‑minute walk test) 5) Physical activity: Revised community Healthy Activities Model Program for Seniors (CHAMPS) questionnaire |
| Eakin et al., 2007 | Low‑income, predominantly Spanish‑speaking urban Latino adults with multiple chronic conditions for which a lifestyle intervention focused on physical activity and diet would be appropriate (N= 200) | **Resources for health intervention** A culturally adapted lifestyle intervention targeting diet and physical activity, including two face‑to‑face self‑management support and community linkage sessions with a health educator, three follow‑up phone calls, and three tailored newsletters | Self-management + Telehealth | Developing personal skills, creating supportive environment, strengthening community action | Socio-ecological model | Hybrid: in‑person sessions plus telephone follow‑up and print materials | Bi-lingual health educator | Community health centre | 2 in‑person sessions; 3 follow‑up calls; 3 newsletters | Culturally adapted and translated into Spanish for an urban, low-income, largely Latino patient population | 1) Reach 2) Efficacy: Changes in dietary behaviour, physical activity, and multilevel support for healthy lifestyles between the groups over the three time points 3) Adoption 4) Implementation 5) Maintenance |
| Ell et al., 2010 | Low-income Hispanic patients with major depression and diabetes (N= 387) | **Multifaceted Diabetes and Depression Program (MDDP)**  Socioculturally adapted collaborative care intervention including problem‑solving therapy and/or antidepressant medication using a stepped‑care algorithm, patient choice of first‑line treatment, structured telephone follow‑up for treatment response, adherence and relapse prevention, plus health‑system navigation support.  Two arms: MDDP and enhanced usual care | Collaboration/integrated care + Telehealth | Developing personal skills, reorienting health services, creating supportive environment | None reported | Hybrid: in‑clinic care plus structured telephone follow‑up | Multidisciplinary collaborative care team (Diabetes depression clinical specialists, assistant patient navigator, psychiatrist) | Public safety‑net primary care clinics | 12 months intervention with outcomes followed to 18 months | Socioculturally adapted for low‑income Hispanic patients. Sociocultural adaptations included psychoeducation and culturally relevant navigation support. | 1) Depression severity (Symptom Checklist‑20, PHQ-9) 2) Health related quality of life (SF‑12) 3) Disability (Sheehan Disability Scale) 4) Anxiety (The Brief Symptom Inventory) 5) Self-reported adherence (Summary of Diabetes Self-Care Activities Questionnaire) 6) Diabetes symptoms (Whitty 9-item questionnaire) 7) Socioeconomic stressors ((financial situation, work, unemployment, financial problems, marital/family conflicts, legal and care-giving problems, and community violence worry). 8) Others (A1C, BMI, comorbidities, antidepressant medication prescription, weight, diabetes complications) |
| Ell et al., 2017 | Adult patients with depression (N= 348) and concurrent chronic condition (diabetes= 96%, and/or heart disease= 4%) | **A-Helping-Hand (AHH)** Two-arm bilingual promotora‑led depression and chronic disease self‑care management program including psychoeducation, support for depression treatment engagement, chronic illness self‑management, care coordination, and navigation of health and community resources. | Task shifting | Developing personal skills, reorienting health services, creating supportive environment, strengthening community action | None reported | In-person | Bilingual Promotoras supervised by the research team, coordinating with clinic providers | Public safety‑net community clinics (Los Angeles County Department of Health Services) | Six weekly psychoeducational sessions plus booster contacts (with assessments at baseline, 6 and 12 months) | Culturally and linguistically tailored for Latino patients with bilingual promotoras providing culturally relevant education | 1) Depression outcomes (PHQ-9 and SCL depression scale) 2) Chronic disease self-efficacy (SEMCD) 3) Patient knowledge, skill, and confidence for self-management (patient activation measure (PAM)) 4) Quality of life (SF-12) 5) Social support (Medical Outcomes Study MOS-SS) 6) Socioeconomic stress (10 items from the Hispanic Stress Inventory modules) 7) Medical care utilisation data (hospitalisation, lab test results, pharmacy pick up records) |
| Feldman et al., 2016 | Latino adults with asthma and panic disorder (N= 53) | **Cognitive‑Behavioural Psychophysiological Therapy (CBPT) C**ulturally adapted intervention integrating CBT for panic disorder, asthma education, training to distinguish panic vs asthma symptoms, and heart‑rate‑variability biofeedback; compared to Music and Relaxation Therapy (MRT) | Self-management | Developing personal skills, reorienting health services, creating supportive environment | Cognitive behaviour therapy | In‑person therapy sessions | The therapists included a clinical psychology post-doctoral fellow and advanced graduate students | Outpatient clinical setting | Eight weekly sessions (with post‑treatment and 3‑month follow‑up) | Cultural adaptation of treatment manuals. Translation and adaptation for instruments that lacked existing Spanish translations with sufficient reliability and validity | **Primary outcomes** 1) Panic disorder severity (The Panic Disorder Severity Scale (PDSS)) 2) Anxiety symptoms (Anxiety Sensitivity Index-3 (ASI-3) 3) Adherence to inhaled corticosteroids medication adherence (Medication 4) Adherence Report Scale (MARS) 5) Asthma control (Asthma Control Questionnaire (ACQ)) 6) Clinical Global Impression Scale (CGI) **Secondary outcomes** 1) Anxiety concerns and symptoms (Agoraphobia Cognitions Questionnaire (AGOR) 2) Body Sensations Questionnaire (BSQ)) 3) Depression measure (Beck Depression Inventory-II (BDI)) 4) Asthma related quality of life (Mini Asthma Quality of Life Questionnaire (AQLQ)) 5) Treatment of asthma and panic disorder (credibility/expectancy questionnaire (CEQ)) |
| Gallo et al., 2023 | Latino adults with multiple cardiometabolic conditions and behavioural health concern (e.g., depressive symptoms, alcohol misuse) (N= 536) | **Mi Puente (My bridge)** Culturally appropriate program of usual care plus inpatient and telephone encounters with behavioural health nurse and community mentor team who addressed participants' social, medical, and behavioural health needs. | Collaborative/Integrated care + Telehealth | Developing personal skills, reorienting health services, creating supportive environment, strengthening community action | Socioecological model | Inpatient hospital visits and telephone calls | Behavioural health nurse and trained community mentors (team‑based model) | Hospital in South San Diego County | Inpatient visit(s) plus structured post‑discharge phone encounters (dose varied by participant needs). Primary outcomes at 30 and 180 days | The intervention adapted processes and materials from evidence-based programs including project Re-Engineered Discharge (Project RED) and the Care Transitions Intervention to the specific socioeconomic, cultural, and health context. | **Primary outcome** 1) Hospital readmissions (30‑ and 180‑day readmissions, ED and observation visits) **Secondary outcomes** 1) Count of readmissions 2) Total inpatient length of stay 3) Patient‑reported outcomes (Patient-Reported Outcomes Measurement Information System (PROMIS®), Global-10 Health Scale, the 13- item Patient Activation Measure (PAM), and a short version of the Chronic Illness Resources Survey (CIRS)) 4) Self-reported healthcare use in the past 3 months 5) Self-reported inpatient days |
| Gardiner et al., 2019 | Predominantly low income racially diverse adults with nonspecific chronic pain and depressive symptoms (N= 159) | **Integrative Medical Group Visits (IMGV)** Group‑based intervention combining mindfulness practices, evidence‑based integrative medicine modalities, and facilitated medical group visits; compared with usual primary care provider (PCP) visits | Collaborative/integrated care + Telehealth | Developing personal skills, reorienting health services, creating supportive environment | None reported | In‑person group sessions and online platform access | Trained physician and non-physician | Academic safety‑net hospital and affiliated federally qualified community health centres | Nine weekly 2.5‑hour in‑person group visits; 12‑week online maintenance phase; final group visit at 21 weeks | None reported | **Primary outcomes** 1) self-reported pain (Brief Pain Inventory (BPI) pain interference 2) depression level (PHQ-9) **Secondary outcomes** 1) Pain self-efficacy (Pain Self-Efficacy Scale (PSEQ))  2) Self-reported pain medication use (Timeline Follow Back method) 3) Health-related quality of life (SF-12) 4) Activation in patients (Patient Activation Measure (PAM)  5) The risk of opioid misuse was (Common Opioid Misuse Measure (COMM)) 6) Self-reported emergency department use |
| Jean-Louis et al., 2017 | Blacks With Metabolic Syndrome and Sleep Apnoea (N= 303) | **MetSO trial: tailored telephone- delivered intervention (TTI)** Culturally and linguistically tailored, telephone‑delivered behavioural intervention using individualised obstructive sleep apnoea education, motivational messaging, and barrier‑focused counselling based on readiness to change; compared with attention‑control | Self-management + Telehealth | Developing personal skills, creating supportive environment, strengthening community action | PRECEDE and the Transtheoretical Model of Change | Telephone | Trained health educators | Virtual | Up to 10 structured telephone calls over the intervention period | The TTI was tailored culturally and linguistically to conform to specific cultural characteristics of black patients identified via focus groups. | 1) Adherence rates for OSA consultation and laboratory- based evaluation as well as positive airway pressure (PAP) treatment |
| Jones et al., 2024 | Adults with HIV and chronic pain (N= 278) | **Skills TO Manage Pain (STOMP)** A behavioural pain self‑management intervention combining individual skill‑building sessions delivered by trained staff with group sessions co‑led by peer interventionists compared with enhanced usual care (manual only) | Self-management + Telehealth | Developing personal skills, creating supportive environment, strengthening community action | Social cognitive theory | 1‑on‑1 sessions plus group sessions (switched to online delivery due to COVID) | Trained staff and peer interventionists | Large academic HIV medical centres (University of Alabama at Birmingham; University of North Carolina–Chapel Hill) | Up to 6 individual sessions and 6 group sessions over approximately 12 weeks. Follow‑up at 3 months | None reported | **Primary** 1) Pain measure (Brief Pain Inventory (BPI) and PEG scale) **Secondary** 1) Pain self-efficacy (Pain Self-Efficacy Questionnaire [PSEQ]) 2) Mood (Patient Health Questionnaire depression scale [PHQ-8]) 3) Pain catastrophising (Pain Catastrophizing Scale [PCS]) |
| Kangovi et al.,2017 | High-poverty neighbourhood residents, uninsured or publicly insured, and diagnosed with ≥2 chronic conditions (diabetes, obesity, hypertension, tobacco dependence) (N= 302) | **Individualised Management for Patient-Centred Targets (IMPaCT)** A CHW intervention that can be applied across diseases to address “upstream” socioeconomic and behavioural barriers. Patients set disease‑management goals with clinicians; intervention group additionally received individualised CHW support addressing social barriers, health behaviours, care navigation, and goal attainment | Task shifting + Telehealth | Developing personal skills, reorienting health services, creating supportive environment, strengthening community action | None reported | Hybrid: In‑person and telephone contacts | Trained community health workers integrated with primary care teams | Outpatient setting | At least once per week for 6. Outcomes assessed up to 12 months | Adapted for outpatients with multiple chronic conditions | **Primary outcome** 1) Mean change in control of patients’ selected chronic disease (HbA1c, BMI, SBP, and self-reported number of cigarettes per day between enrolment and 6-month follow-up) **Secondary outcomes** 1) (yes or no) of chronic disease management goals 2) mean change in self-rated health (SF-12) 3) Patient activation measure 4) Proportion of patients reporting high quality of patient-centred care (as measured by the “comprehensiveness of care” and “support for patient self-management” domains of the Consumer Assessment of Healthcare Providers and Systems–Patient Centered Medical Home [CAHPS-PCMH] survey 5) All-cause hospitalisations (at 6 and 12 months) |
| Lynch et al., 2014 | Low-income African American adults with type 2 diabetes and hypertension (N= 61) | **Lifestyle Improvement Through Food and Exercise (LIFE)** An intensive group‑based program focused on diet, physical activity, behavioural skills training, and peer support, supplemented by weekly peer‑support telephone calls; compared with brief diabetes self‑management education | Self-management + Telehealth | Developing personal skills, creating supportive environment, strengthening community action | Cognitive behavioural models of behaviour change | In‑person group sessions plus telephone follow‑up | Registered dietitian (group sessions) and trained peer supporter (calls) | Community setting | 18 group sessions over 6 months plus weekly peer‑support phone calls | Culturally tailored education for African Americans | **Primary outcome** 1) 5% weight reduction at 6 months **Secondary outcome** 1) 0.5 percentage-point reduction in HbA1c |
| Metsch et al., 2016 | Hospitalised adults with HIV infection and substance use (N= 801) | **Hospital Visit as Opportunity for Prevention and Engagement for HIV-infected Drug Users (Project HOPE)** **Patient Navigation (PN**) Structured care coordination including case management and motivational interviewing **PN + Financial Incentives** PN plus contingent cash incentives (up to $1,160) for achieving predefined care and substance‑use–related behaviours | Collaborative/integrated care | Developing personal skills, reorienting health services, creating supportive environment, strengthening community action | None reported | In-person | Trained patient navigators | Inpatient hospital setting | Up to 11 navigation sessions delivered over 6 months. Outcomes assessed at 6 and 12 months | None reported | 1) The primary outcome was HIV viral suppression (defined as having a viral load of ≤200 copies/mL) versus HIV viral non-suppression or death at 12 months. |
| Metsch et al., 2021 | Adults with HIV and hepatitis C virus (HCV) coinfection and active substance use (N= 113) | **Care Facilitation intervention** Structured, motivation‑ and strengths‑based case management focused on retrieving HCV viral load results, engagement in HIV/HCV care, appointment facilitation, and medication adherence; compared with referral to HCV evaluation and assistance with scheduling | Collaborative/integrated care | Developing personal skills, reorienting health services, creating supportive environment, strengthening community action | None reported | Hybrid: in-person or over the phone | Trained care facilitators | In-patient setting | Approximately twice each month for 12 months | None reported | 1) The primary outcome was number of steps achieved along a series of 8 potentially nonsequential clinical steps of the HCV/HIV care continuum over 12 months post randomization. **Secondary outcomes** 1) Proportion of participants who had at least 1 HIV care visit across the follow-up period 2) HIV viral suppression 3) In-patient hospitalisation 4) self-reported substance use 5) Participation in substance use treatment 6) Urine drug screen |
| Nuño et al., 2023 | Hispanic adults with type‑2 diabetes, at high risk for cardiovascular disease (N= 30) | **Meta Salud Diabetes (MSD)** A culturally appropriate behavioural health and lifestyle intervention addressing diabetes and cardiovascular disease risk; covers nutrition, physical activity, chronic disease education, and behavioural risk reduction | Self-management | Developing personal skills, reorienting health services, creating supportive environment, strengthening community action | None reported | In-person | *Promotoras from Federally Qualified Health Centres* | Federally Qualified Health Centres | 2h class sessions delivered over a 13-week period | culturally tailored for Hispanic population | **Implementation science outcomes** 1) Feasibility: recruitment rates, retention rates, participants feedback **Clinical outcomes**  1) Framingham Risk Score (FRS) 2) Haemoglobin A1c (Hb1c) 3) Fasting blood glucose 4) blood pressure (systolic and diastolic)  Triglycerides total cholesterol high-density lipoprotein cholesterol (HDL), and low-density lipoprotein cholesterol (LDL).  5) Anthropometric measures were (body mass index (BMI), weight, waist, hip, and height)  6) Health-Related Quality of Life (HRQoL) 7) Emotional distress after t2D diagnosis (The Problem Areas in Diabetes Questionnaire (PAID)) |
| Ogedegbe et al., 2024 | Black and Hispanic adults with prior stroke and uncontrolled hypertension (N= 450) | **Home Blood Pressure Telemonitoring (HBPTM) plus Nurse Case Management (NCM)** HBPTM with wireless transmission of BP data combined with structured, telephone‑based nurse counselling for medication management, lifestyle modification, and care coordination; compared with HBPTM alone | Task shifting + Telehealth | Developing personal skills, reorienting health services, creating supportive environment | None reported | Home BP telemonitoring with remote data transmission plus telephone nurse coaching | Trained nurse case managers | Virtual | HBPTM: 12 BP measurements per week for 12 months NCM: up to 20 structured counselling calls over 12 months 12 months of active intervention with stroke recurrence follow‑up through 24 months | None reported | **Primary outcomes** 1) within-patient change in SBP from baseline to 12 months (mean of 3 BP readings) 2) rate of recurrent stroke at 24 months **Secondary outcomes** 1) 12- and 24-month cost-effectiveness **Post-hoc outcomes** 1) Mortality as well as other vascular events, including heart attack and heart failure |
| Rimmer et al., 2002 | African American, Asian American, Hispanic, White with type 2 diabetes (N= 30) and high blood pressure (75.9%) | **Structured health promotion intervention G**roup‑based program focusing on diet, nutrition education, physical activity, and health behaviour change, with logistical supports (e.g., free transportation) to reduce participation barriers | Self-management | Developing personal skills, creating supportive environment | None reported | In-person group sessions | Multidisciplinary program staff (health promotion and nutrition professionals, registered nurse) | University-based | 3 pre-screening visits, 12‑week structured group intervention | None reported | 1) Blood measures 2) Exercise measures: symptom-limited graded exercise test (peak VO2), heart rates, strength 3) Nutrition measures: Rate your plate eating pattern assessment, diabetes self-efficacy scale, 1 week food frequency questionnaire 4) Health behaviour: life satisfaction questionnaire, CES-D |
| Shah et al., 2024 | South Asian immigrants with type 2 diabetes and comorbid hypertension (N= 190) | **DREAM Atlanta intervention (Diabetes Research, Education, and Action for Minorities)** Culturally tailored, CHW‑led telehealth program delivering group education on diabetes and hypertension, individualised action planning, and follow‑up goal‑setting calls; compared with minimal‑dose control | Task shifting + Telehealth | Developing personal skills, reorienting health services, creating supportive environment, strengthening community action | Health Belief Model and Social Support Theory | Telehealth (virtual group sessions and follow‑up phone calls) | Trained community health workers from South Asian communities | Virtual | Five virtual group education sessions (lasting approximately 60 minutes) plus follow‑up goal‑setting calls over ~6 months | Culturally and linguistically adapted | **Primary outcome** 1) Change in BP control, defined as < 130/80 mmHg, between baseline and 6-month study endpoint. **Secondary clinical outcome** 1) SBP, mmHg, DBP, mmHg, haemoglobin A1c (HbA1c, %), weight (lbs.), and body mass index (BMI, km2). **Feasibility outcome** 1) Recruitment and enrolment metrics (i.e., proportion of recruited participants who enrol) 2) Proportion of participants who complete all sessions, and baseline and follow-up survey completion. **Patient-centred outcome**  1) Self-reported physical activity (culturally modified from the Behavioural 2) Risk Factor Surveillance System [BRFSS]) 3) Daily diet intake (culturally modified from the BRFSS) 4) Medication adherence (Adherence to Refills and Medications Scale [ARMS]), Diabetes self-management 5) Diabetes physician management 6) Health self-efficacy (adapted from the Bandura self-efficacy scale) 7) Depression risk (Patient Health Questionnaire-224 [PHQ-2]) 8) Instrumental support (NIH toolbox) 9) Days of poor physical and mental health (BRFSS) |
| Tobe et al., 2006 | First Nations adults with existing hypertension and type 2 diabetes. (N= 100) | **Nurse‑directed community‑based hypertension management**  Home care nurses implemented a predefined pharmacologic antihypertensive treatment algorithm with regular BP monitoring; compared with usual physician‑directed care | Task shifting | Developing personal skills, reorienting health services, creating supportive environment | None reported | In-person | Home care nurses and home health aides | Community screening clinics | All participants were seen by a home care nurse in the medical clinic on their reserve at baseline, at 6 weeks and at 3,6, 9 and 12 months after enrolment. | None reported | **Primary outcome** 1) Difference between the 2 groups in the change in systolic blood pressure after 12 months.  **Secondary outcomes** 2) Change in diastolic blood pressure over time 3) Change in urine albumin status 4) Incidence of adverse events |
| Towfighi et al., 2021 | Adults ≥40 years with recent ischemic or haemorrhagic stroke or transient ischemic attack (≤90 days) and uncontrolled hypertension (N= 487) | **SUCCEED intervention (Secondary Stroke Prevention by Uniting Community and Chronic Care Model Teams Early to End Disparities)** A multicomponent intervention combining a community health worker (CHW), an advanced practice clinician (nurse practitioner or physician assistant), and physician oversight to address stroke risk factors, self‑management, care coordination, and social barriers; compared with usual care | Collaborative/integrated care | Developing personal skills, reorienting health services, creating supportive environment, strengthening community action | Health Belief Model | In-person- one to one and workshops | Community health workers, advanced practice clinicians, and physicians | Safety‑net hospitals and home visits in Los Angeles County | At least 3 APC clinic visits; at least 3 CHW home visits; telephone calls, self-management workshops | None reported | 1) The primary outcome was change in systolic BP at 12 months. 2) Secondary outcomes were non–high density lipoprotein cholesterol, haemoglobin A1c, and C-reactive protein (CRP) levels, body mass index, antithrombotic adherence, physical activity level, diet, and smoking status at 12 months. |
| Williams et al., 2012 | Adults (≥18 years) with chronic kidney disease, diabetes, and cardiovascular disease from culturally and linguistically diverse (CALD) groups (Greek, Italian, Vietnamese speakers) (N= 78) | **Translated multifactorial intervention** Included a structured medication review, a brief translated educational PowerPoint session, and motivational interviewing designed to improve medication self‑efficacy and adherence; compared with control/usual care | Self-management + Telehealth | Developing personal skills, reorienting health services, creating supportive environment | Health belief model | Hybrid: In-person and telephone | Intervention nurse | Nephrology outpatient clinics in two metropolitan hospitals | 20-minute PowerPoint presentation, and fortnightly follow-up telephone motivational interviewing for 12 weeks | Intervention materials translated into translated into Greek, Italian and Vietnamese | **Primary outcomes** 1) Medication self-efficacy: The 26-item Medication Adherence Self-Efficacy Scale (MASES) 2) Medication adherence: The four-item Morisky medicine adherence scale  **Secondary outcomes** 1) SF-12 2) Health care use: Lorig et al.’s (1996) four-question health care utilisation scale 3) Routine clinical measures |
| Zuniga et al., 2019 | Adults (≥18 years) living with HIV and type 2 diabetes (N= 25) | **Adapted diabetes self‑management educational intervention for PLWH** A community‑based diabetes self‑management program adapted for people living with HIV, consisting of structured diabetes education plus follow‑up support calls | Self-management + Telehealth | Developing personal skills, reorienting health services | None reported | Hybrid: In-person and telephone | Trained interventionist, registered nurse or licensed social worker | Federally Qualified Health Centres (FQHCs) HIV clinics | 12 weeks long with 6h of educational instruction followed by 6 weekly support telephone calls | Adapted from Kim et al.’s community-based self-management intervention for diabetes | 1) Adherence to medication treatment  2) Diabetes self-management activities (Summary of Diabetes Self-Care Activities scale (SDSCA)) 3) Diabetes knowledge (Diabetes Knowledge Test (DKT))  4) HIV knowledge (HIV Knowledge Questionnaire (HIVKQ)) |
